# Alloying Motif Confined in Intercalative Frameworks toward Rapid Li‐Ion Storage

**DOI:** 10.1002/advs.202202026

**Published:** 2022-06-17

**Authors:** Xueyu Lin, Chenlong Dong, Siwei Zhao, Baixin Peng, Ce Zhou, Ruiqi Wang, Fuqiang Huang

**Affiliations:** ^1^ Beijing National Laboratory for Molecular Sciences and State Key Laboratory of Rare Earth Materials Chemistry and Applications College of Chemistry and Molecular Engineering Peking University Beijing 100871 P. R. China; ^2^ State Key Laboratory of High Performance Ceramics and Superfine Microstructure Shanghai Institute of Ceramics Chinese Academy of Sciences Shanghai 200050 P. R. China

**Keywords:** alloy, anodes, intercalative frameworks, lithium‐ion batteries, metal oxide

## Abstract

High‐capacity alloying‐type anodes suffer poor rate capability due to their great volume expansion, while high‐rate intercalation‐type anodes are troubled with low theoretical capacity. Herein, a novel mechanism of alloying in the intercalative frameworks is proposed to confer both high‐capacity and high‐rate performances on anodes. Taking the indium‐vanadium oxide (IVO) as a typical system, alloying‐typed In is dispersed in the stable intercalative V_2_O_3_ to form a solid solution. The alloying‐typed In element provides high lithium storage capacity, while the robust, Li‐conductive V−O frameworks effectively alleviate the volume expansion and aggregation of In. Benefiting from the above merits, the anode exhibits a high specific capacity of 1364 mA h g^−1^ at 1 A g^−1^ and an extraordinary cyclic performance of 814 mA h g^−1^ at 10 A g^−1^ after 600 cycles (124.9 mA h g^−1^ after 10 000 cycles at 50 A g^−1^). The superior electrochemical rate capability of (In,V)_2_O_3_ solid solution anode rivals that of the reported alloying anode materials. This strategy can be extended for fabricating other alloying/intercalation hybrid anodes, such as (Sn,V)O_2_ and (Sn,Ti)O_2_, which demonstrates the universality of confining alloying motifs in intercalative frameworks for rapid and high‐capacity lithium storage.

## Graphical abstract

A universal model of high capacity and high‐rate anodes containing well‐spread alloying‐type element in the robust intercalation‐type framework is proposed. Representative (In,V)_2_O_3_ solid solution anode delivers high capacity of 1364 mA h g^−1^ at 1 A g^−1^ and an extraordinary cyclic performance of 124.9 mA h g^−1^ after 10 000 cycles at 50 A g^−1^.

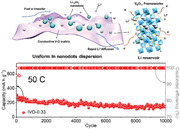

## 1 Introduction

**Figure 1:**
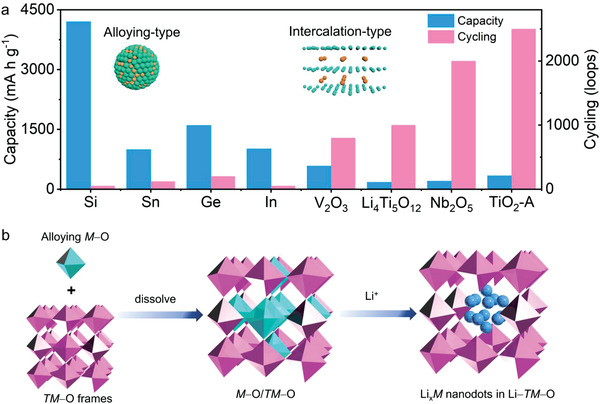
a) Comparison of theoretical capacity and cycling among typical intercalation‐type and alloying‐type anodes.^[^ ^2^, ^14^, ^15^, ^16^, ^17^, ^18^, ^19^, ^20^ ^]^ b) Schematic illustration of dispersing fragile alloying‐type *M*−O into a robust intercalation‐type *TM*−O frameworks to form an intercalation/alloying combined structure. After fully lithiation Li*_x_M* nanodots (deep blue sphere) could be confined in Li−*TM*−O frameworks.

High‐performance lithium‐ion battery (LIBs) anode combining high capacity and stable rate capability is critical for the fabrication of high energy‐density and power‐density devices. Alloying‐type anodes are good candidates to build high‐energy‐densities devices due to their high theoretical capacities (Si: 4200 mA h g^−1^, Ge: 1600 mA h g^−1^, Sn: 992 mA h g^−1^, In: 1010 mA h g^−1^, etc.),^[^ ^1^, ^2^, ^3^, ^4^, ^5^ ^]^ while these materials still suffer severe volumetric change (250–420%), and the consequent poor cyclability especially in high‐rate conditions, as summarized in **Figure** **1**a; and Figure S1 (Supporting Information).^[^ ^2^, ^6^ ^]^ By comparison, intercalation‐type anodes possess rapid electrochemical reactivity with negligible volumetric change (<1%) for high‐rate charging/discharging, while their theoretical capacities are relatively low (TiO_2_: 335 mA h g^−1^, Li_4_Ti_5_O_12_: 175 mA h g^−1^, T‐Nb_2_O_5_: 200 mA h g^−1^, etc.).^[^ ^7^, ^8^, ^9^, ^10^ ^]^ Therefore, it remains a big challenge to design an anode paradigm which can combine the two critical performances simultaneously.

The different lithium‐storage characters of alloying and intercalation‐type metal oxides origin from the distinction in their bonding affinities toward O. The capability of metal oxides (*M*O_n_) for Li^+^ uptake and extraction can be evaluated by the relative electromotive force (EMF, EMF = −*∆G/nF*) obtained from the following reaction *2n*Li^+^+ *M*O_n_ +*2n*e^−^ = *M* + *n*Li_2_O. Higher EMF value (usually > 1 V vs Li^+^/Li) indicates a complete Li^+^ uptake but a poor Li^+^ extraction, while lower EMF value (< 1 V vs Li^+^/Li) means an insufficient Li^+^ uptake but a complete Li^+^ extraction.^[^ ^11^ ^]^ As shown in Figure S2a (Supporting Information), due to relatively weak M−O bonds in the alloying‐type metal oxides, the Gibbs free energies (*∆G*) of the reactions are quite negative, resulting in large EMFs. The intercalation‐type metal oxides with strong M−O bonds always possess small EMFs. As the ideal anodes with high capacity and stable rate capability demands both complete Li^+^ uptake and Li^+^ extraction reaction, a moderate EMF value should be critical, which can be modified by tuning the bonding energy of M−O in the metal oxides.

In aim to regulate the M−O bonding energy for the optimized EMF value, a bi‐metal‐oxide model which confines alloying motif in intercalative frameworks was design. In this model, alloying‐type elements (Si, Ge, Sn, In, and Sb) are uniformly dispersed in the stable intercalation‐type frameworks (TiO_2_, V_2_O_3_, and Nb_2_O_5_, Figure 1b). Alloying‐type elements offer extra Li^+^ storage at significantly lower potential compared to typical intercalation‐type anode (Figure S2b, Supporting Information), contributing to a larger capacity with decreased overall potential. The intercalation‐type frameworks can provide a buffering effect for the alloying‐type anode to relieve the volume expansion. Therefore, it is beneficial to combine the advantages and minimize the demerits of the intercalation/alloying mechanism in this model simultaneously. The anode systems of Sn/TiO_2_^[^ ^12^ ^]^ and Ce/SnO_2_,^[^ ^13^ ^]^ we previously reported are preliminary attempts of this strategy, which gave experimental supports to its effectiveness.

**Figure 2:**
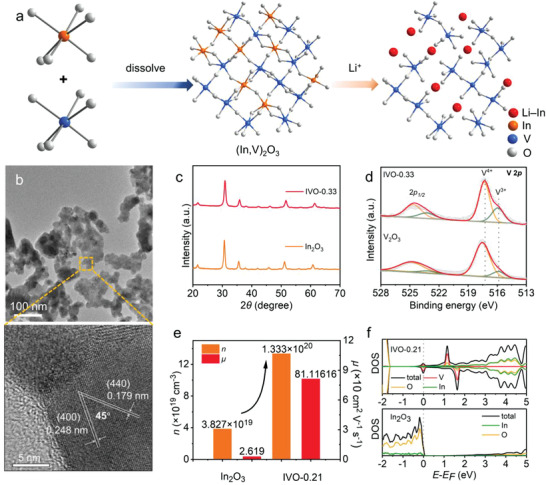
a) Schematic illustration of constructing an intercalation/alloying mechanism combined (In,V)_2_O_3_ solid solution. b) TEM image and HRTEM image of IVO‐0.33. c) XRD pattern of IVO‐0.33 and In_2_O_3_. d) XPS spectra of IVO‐0.33 and V_2_O_3_. e) Comparison of carrier concentration and carrier mobility for IVO‐0.21 and In_2_O_3_. f) Electronic density of states of cubic IVO‐0.21 and In_2_O_3_.

Herein, we designed a bi‐metal oxide solid solution (In,V)_2_O_3_ with atomically dispersed bifunctional components. In_2_O_3_ is a classical conversion‐alloying anode with high theoretical capacity of 1415 mA h g^−1^ (based on In_2_O_3_ start material), while V_2_O_3_ possesses intercalative behavior. Both In_2_O_3_ and V_2_O_3_ can adopt the Bixbyite‐type structure with the space group of *Ia* $\bar{3}$.^[^ ^21^ ^]^ In addition, In^3+^ and V^3+^ ions show similar ion radii (0.94 and 0.80 Å, respectively) and coordination, which facilitates the bi‐metal oxide solid solution via simple synthetic process. Each component in this solid solution exhibits individual function. Conversion‐alloying‐type In_2_O_3_ (3.5–3.7 eV) provides extensive capacity by forming Li_4.33_In/Li_2_O after lithiation because of lower In−O bond energy (346 kJ mol^−1^). Besides, the lithium‐extraction potential of In (≈1.0 V vs Li^+^/Li) is relative lower than typical vanadium oxide (*≈*3.50 V vs Li^+^/Li for V_2_O_5_, 2.73 V vs for VO_2_, 1.65 V for V_2_O_3_), resulting in a decreasing overall voltage of battery (Figure S2b, Supporting Information). Intercalation‐type V_2_O_3_ functions as robust framework due to stable V−O bonding (637 kJ mol^−1^), which could be transformed to hollow Li−V−O motifs with abundant tunnels after lithiation, facilitating the Li^+^ diffusion. After lithiation, the uniform distribution of Li‐In nanodots are confined in a stable conductive Li−V−O matrix, so the volume expansion of indium is hindered (**Figure** **2**a). Based on above considerations, (In,V)_2_O_3_ solid solution nanoparticles were synthesized via solvothermal method. Served as anode for LIBs, (In,V)_2_O_3_ displays remarkable performance of rapid lithium‐capacity storage, including ultrahigh rate capability (124.9 mA h g^−1^ at 50 A g^−1^ over 10 000 cycles) and high capacity of 1364 mA h g^−1^ at 1 A g^−1^ (814 mA h g^−1^ at 10 A g^−1^). The superior electrochemical performance ranks top among previous reported works of In‐based anode. Confining alloying motif in intercalative frameworks provides an effective route to design novel anodes with high capacity and rate capability.

## 2 Results and Discussion

### 2.1 Synthesis and Characterization of (In,V)2O3 Solid Solution

To achieve better lithium storage performance, nanosized (In,V)_2_O_3_ solid solution with tunable In/V ratios were successfully synthesized via solvothermal method.^[^ ^22^, ^23^, ^24^ ^]^ Blue‐gray IVO‐*x* (*x* was defined as vanadium atomic percentage, *x* = 0.25, 0.33, 0.5, 0.66, and 0.83, Figure S3, Supporting Information) samples were obtained from V_2_O_5_, indium acetate and ethanol through a facile reduction solvothermal method followed by treatment. V_2_O_5_ is known as a typical catalyst for alcohol oxidation.^[^ ^25^, ^26^ ^]^ During the solvothermal procedure, the ethanol was oxidized accompanying the reduction of V^V^. To further promote the introduction of V to In_2_O_3_ lattice, a treatment in argon atmosphere was performed on solvothermal product. V_2_O_3_ and In_2_O_3_ were also prepared in similar ways for rational comparison. The ratio of In and V in these samples were analyzed by ICP‐AES (Table S1, Supporting Information). From the ICP results, the actual elemental concentration of In and V in IVO‐*x* was consistent with the nominal composition of (In,V)_2_O_3_ solid solution.

The morphology and microstructure of IVO‐0.33 is further characterized by scanning electron microscopy (SEM) and transmission electron microscopy (TEM). Different from the sheets‐assembled morphology of V_2_O_3_, In_2_O_3_, and IVO‐0.33 are assembled from nanoparticles of ≈30 nm (Figure 2b; and Figure S4a–d, Supporting Information). To verify the microstructure, high resolution TEM (HRTEM, Figure 2b; and Figures S4e and S5a, Supporting Information) is performed. The HRTEM images display lattice fringes of 0.291, 0.251, and 0.179 nm, corresponding to (222), (400), and (440) plane of cubic In_2_O_3_, respectively. The measured angle between (400) and (440) plane is 45°, which is similar with the theoretical value of 45.8°. The corresponding selective area electron diffraction (SAED, Figure S4f, Supporting Information) also exhibit the interplane of (211), (222), (400), (440), and (622), showing a good crystalline of IVO‐0.33. No amorphous areas or lattice fringes of VO*_x_* are detected. The high angle annular dark field/scanning transmission electron microscopy (HAADF‐STEM) and elemental mapping show a good elemental distribution of In, V and O (Figure S5b, Supporting Information).

The crystal structures of the obtained nanoscale solid‐solutions are clarified via the powder X‐ray diffraction (PXRD) patterns (Figure 2c). Pure Bixbyite‐type (*Ia* $\bar{3}$) In_2_O_3_ was obtained under the hydrothermal condition. This phase maintained once V is added into the system forming (In,V)_2_O_3_ solid solutions until the V content exceeds 50% (Figure S6a, Supporting Information) Compared with In_2_O_3_, diffraction peaks of IVO‐*x* slightly shift to higher degree with the increasing V substitution up to *x* = 0.5, implying the decrease lattice constant due to smaller V^3+^ ion (0.78 nm) compared with In^3+^ (0.94 nm). When *x* = 0.66, vague peaks at 33.0°, 41.2°, and 53.9° appeared, which could be assigned to *R* $\bar{3}$ *c* phase V_2_O_3_ (Figure S6b, Supporting Information). When *x* = 0.83, In_2_VO_5_ is obtained and no obvious peak of (In,V)_2_O_3_ solid solution can be observed. To further investigate the structure, Rietveld refinements of IVO‐*x* (*x* = 0.25, 0.33, 0.5, and 0.66) and In_2_O_3_ are carried out (Figures S7, S8 and Tables S2–S6, Supporting Information). The lattice parameters of IVO‐0.33 are 10.0157 Å, which are smaller than In_2_O_3_ (10.1120 Å), proving the successful introduction of V into In_2_O_3_ lattice. Figure S9 (Supporting Information) summarizes the phase, composition and the refined lattice parameters of IVO‐*x*, In_2_O_3_, and V_2_O_3_, where the lattice constant (*a*) fits the Vegard's law approximately up to *x* = 0.5.

The valance states of elements in the solid solution are identified by X‐ray photoelectron spectroscopy (XPS). The V 2*p* spectrum (Figure 2d) of both IVO‐0.33 and V_2_O_3_ specimen exhibit two peaks at 517.3 (V 2*p*_3/2_) and 524.5 eV (V 2*p*_1/2_), which can be deconvoluted into +3 and +4 valence state.^[^ ^27^ ^]^ The atomic ratio of V^III^ and V^IV^ at surface is determined to be 1.2 by integrating the area of peaks. Raman spectra are also collected to further understand the structure (Figure S10, Supporting Information). For V_2_O_3_, the band at 139 cm^−1^ can be assigned to the bending of V−O−V bonding. The peaks at 88, 187, and 276 cm^−1^ can be assigned to V−O bending. The peaks at 405 and 501 cm^−1^ can be assigned to the vibration of V−O−V bonding. The peak at 997 cm^−1^ can be assigned to the V−O vibration.^[^ ^28^ ^]^ For In_2_O_3_, Raman peaks at 121, 274, 287, and 478 cm^−1^ can be assigned to A_1g_, T_g_, A_2g_, and A_3g_ mode, respectively.^[^ ^29^ ^]^ For IVO‐0.33, except for the Raman active modes (A_1g_+T_g_+ A_2g_) of In_2_O_3_, the V−O bending (187 cm^−1^) and V−O vibration (905 cm^−1^) can be observed. V−O−V bending is no observed in IVO‐0.33, implying the deficiency of isolated vanadium oxides. Ultraviolet photoemission spectrum (UPS, Figure S11a, Supporting Information) and XPS‐valence band spectra (XPS‐VB, Figure S11b, Supporting Information) are carried out to determine the position of the valance band maximum (*E*_VBM_) with respect to Fermi level (*E*_F_). The *E*_VBM_ with respect to *E*_F_ for In_2_O_3_ and IVO‐0.33 is determined to be 2.18 and 0.2 eV, respectively. Based on secondary electron cut‐off in UPS, work function (*ϕ*) for IVO‐0.33 and In_2_O_3_ is determined to be 4.60 and 4.20 eV, respectively.

To further investigate the effect of V toward the electron transport properties of In_2_O_3_, In_1.581_V_0.419_O_3_ (IVO‐0.21, 0.21 represents the vanadium atomic percentage) was directly prepared by high‐temperature solid‐state reaction of V_2_O_3_ and In_2_O_3_. The XRD pattern can be well refined using the Bixbyite‐type structure (*Ia* $\bar{3}$, *R* = 2.50%, Figures S12, S13 and Tables S7, S8, Supporting Information) without obvious extra peaks, indicating the formation of solid solution. To further study the electronic transport properties of IVO‐0.21, Hall effect of IVO‐0.21 and In_2_O_3_ are measured using Physical Properties Measurement System (PPMS, Figure 2e; and Figure S15, Supporting Information). The positive Hall resistivity (*R*_H_) values of IVO‐0.21 and In_2_O_3_ revealed *n*‐type conductivity. Due to the incorporation of V, IVO‐0.21 possesses larger carrier concentration (1.33 × 10^20^ cm^−3^) compared with that of In_2_O_3_ (3.83 × 10^19^ cm^−3^),^[^ ^30^ ^]^ as well as higher carrier mobility (81.12 cm^2^ V^−1^ s^−1^) than that of In_2_O_3_ (2.62 cm^2^ V^−1^ s^−1^), accelerating mobilities of the charge carriers with increasing concentration realized. Therefore, an enhanced electronic conductivity for the (In,V)_2_O_3_ could be observed. Density functional theory (DFT) calculations are carried out to better understand the electronic structure (Figure 2f). The introduction of V 3*d* orbitals lower the conducting band (CB), leading to an overlap between Fermi level and CB, accelerating the electron transfer in IVO solid solution. From the DFT, XPS‐VB, and UPS results, the introduction of V generates an impurity level, implying a metallic state in (In,V)_2_O_3_ different from the semiconducting state of In_2_O_3_.

### 2.2 Li‐Ion Storage Performance

The electrochemical behaviors of IVO‐0.33 for lithium storage are first analyzed by cyclic voltammetry (CV, Figure S16a–c, Supporting Information). The cathode peaks at 0.633 and 0.495 V (vs Li^+^/Li) in the 1st cycle can be attributed to the lithiation of IVO‐0.33 to form the In and the multisteps alloying reactions of In, respectively. The anode peaks at 0.703, 1.203, 1.720 V (vs Li^+^/Li) can be ascribed to the multistep Li‐extraction of Li_4.33_In, Li_3.5_In (composed of Li_4_In and Li_3_In), and Li_1.75_In (composed of Li_2_In and LiIn), respectively.^[^ ^4^ ^]^ For In_2_O_3_, the anodic peak at ≈0.703 V could be assigned to Li‐extraction of Li_4.33_In to form Li_3.5_In, which is a thermodynamic process, while the anodic peak at 1.720 V could be assigned to the Li‐extraction of Li_3.5_In, which is controlled by the diffusion rate of Li^+^.^[^ ^4^ ^]^ For IVO‐0.33, the subsequent Li‐extraction of Li_3.5_In occurs at 1.203 V, indicating thorough extraction of Li_3.5_In in IVO‐0.33 with a higher Li^+^ diffusion rate. Compared with In_2_O_3_, the narrowed potential separation between anode and cathode peaks in IVO‐0.33 could be assigned to better electrochemical reactivity and smaller polarization. CV curve of V_2_O_3_ reveal the pseudocapacitive behavior, quite different from In_2_O_3_. The CV curves of IVO‐0.33 and V_2_O_3_ in the range of 0–3 V exhibits relatively high reversibility suggesting the advantages in the V−O matrix for In dispersing. To quantify the capacity contribution from the Li‐extraction of Li_4.33_In and Li_3.5_In/Li_1.75_In in IVO‐0.33 and In_2_O_3_, charge capacity at 0.1 A g^−1^ is divided by the potential range of 0–0.8 and 0.8–3 V (Figure S16d, Supporting Information). At the potential range of 0.8–3 V (corresponding to the Li‐extraction of Li_3.5_In/Li_1.75_In), IVO‐0.33 generated a charge capacity of 1400 mA h g^−1^ after 100 cycles, while In_2_O_3_ generates a capacity of 700 mA h g^−1^. At the potential range of 0–0.8 V (corresponding to the Li‐extraction of Li_4.33_In to form Li_3.5_In), the capacity of IVO‐0.33 is approximately equal to the counterpart of In_2_O_3_. Thus, the increment of capacity in (In,V)_2_O_3_ occurred at a potential range of 0.8–3 V, which could be attributed to the accelerating kinetics and improved reaction reversibility in IVO‐0.33.

The increment of capacity from the Li‐extraction of Li_3.5_In/Li_1.75_In proves the excellent Li storage performance of IVO‐0.33. From Figure S16f (Supporting Information), after 100 cycles, IVO‐0.33 displays a capacity of 2028 mA h g^−1^ with the initial coulombic efficiency (ICE) of 73.9% at 0.1 A g^−1^, while In_2_O_3_ and V_2_O_3_ displays a capacity of 1278 mA h g^−1^ (ICE: 67.7%) and 358 mA h g^−1^ (ICE: 61.5%), respectively. The molar amount of Li^+^ stored in per formula unit of active material are quantified by the equation *C* = *nF*/3.6*M*, where *C* is the capacity, *n* is the number of reactive electrons (equal to number of Li^+^ accommodated in lattice host), *F* is Faradic constant, and *M* is the Molar weight.^[^ ^41^ ^]^ In 100th cycle, the Li‐extraction/intercalation amount in IVO‐0.33 is 17.7/18.2 mol per mole of active material, while the corresponding amount of In_2_O_3_ and V_2_O_3_ were 13.0/13.2 and 2.0/2.0, respectively. The results revealed that a synergistic effect was constructed in IVO‐0.33 to store more Li^+^. It is worth mentioning that 13 mol of Li^+^ could be stored per formula unit for In_2_O_3_ and closed to theoretical value of 14.66, which could be benefited from short diffusion path with improved kinetics of nanosizing.^[^ ^22^ ^]^

**Figure 3:**
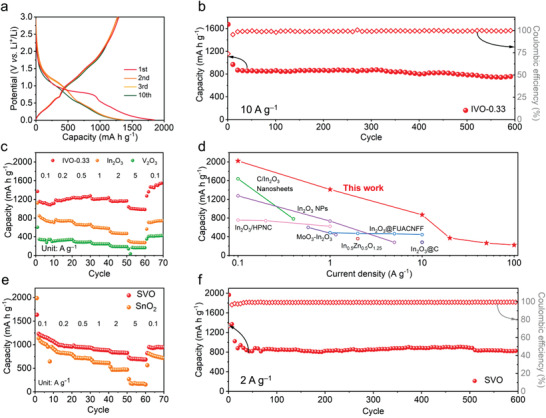
Li‐ion storage performance. a) Charge–discharge curves of IVO‐0.33 at 1st, 2nd, 3rd, and 10th cycle. b). Cycling performance of IVO‐0.33 at a current density of 10 A g^−1^ (First 10 cycles: 0.1 A g^−1^). c) Rate capability of IVO‐0.33 at a current density of 0.1, 0.2, 0.5, 1, 2, 5 A g^−1^. d) Li‐storage performance comparison among IVO‐0.33 and previously reported In‐based anodes.^[^ ^18^, ^31^, ^32^, ^33^, ^34^, ^35^, ^36^, ^37^, ^38^, ^39^, ^40^ ^]^ e) Rate capability of SVO and SnO_2_ at 0.1, 0.2, 0.5, 1, 2, 5 A g^−1^. f) Long cycle performance of SVO at 2 A g^−1^.

The effect of V concentration in (In,V)_2_O_3_ on cyclic performance is also investigated by using synthesized (In,V)_2_O_3_ solid solution with different atomic ratio of In/V. (In,V)_2_O_3_ with different In/V ratio displays similar tilting charge/discharge platform (**Figure** **3**a; and Figure S17a, Supporting Information). The charge mean voltage of In_2_O_3_, IVO‐0.25, IVO‐0.33, IVO‐0.5, IVO‐0.66, and V_2_O_3_ are 1.07, 1.26, 1.21, 1.22, 1.20, and 1.81 V (vs Li^+^/Li), respectively (Figure S19 and Table S9, Supporting Information). Compared to V_2_O_3_, the introduction of In lower the discharge/charge mean voltage. It is noted that V_2_O_3_ did not display any obvious charge/discharge plateau, implying the capacitive charge storage mechanism with rapid electronic and ionic transport kinetics, which is common in other V_2_O_3_ reports served as LIBs anode,^[^ ^42^, ^43^, ^44^ ^]^ cathode for Mg‐ion batteries^[^ ^45^ ^]^ and electrode for Li‐ion capacitor.^[^ ^46^ ^]^ Figure S17b (Supporting Information) displays the cyclic performance of (In,V)_2_O_3_ with various In/V ratio at 0.1 A g^−1^. All the (In,V)_2_O_3_ display stable cyclic performance, where IVO‐0.33 exhibits the highest capacity with a moderate ICE of 73.9%, while IVO‐0.66 delivered highest ICE of 76.2% but lowest capacity due to the smallest proportion of In. (Table S9 and Figure S17b, Supporting Information).

Cyclic performance of IVO‐0.33 and In_2_O_3_ at 1 A g^−1^ (Figure S18, Supporting Information) are performed. The capacity increases in initial cycles and reaches a maximum of 1478.9 mA h g^−1^ at 140th cycle. After 500 cycles, a reversible capacity of 1364 mA h g^−1^ can be achieved in IVO‐0.33. To rational compare, the capacity of In_2_O_3_ remains at 742.9 mA h g^−1^ after 260th cycles at 1 A g^−1^. The increasing capacity of IVO‐0.33 can be attributed to the capacitive contribution of newly formed capacitive V—O frames. The tilting charge/discharge platforms of IVO‐0.33 reveal the pseudocapacitive Li^+^ storage behavior (Figure S18a, Supporting Information). Compared with In_2_O_3_ and In_2_O_3_/C, IVO‐0.33 displays an excellent cycling stability and prominent capacity at large current densities (Figure 3b; and Figures S20 and S21, Supporting Information). At a current density of 10 A g^−1^, IVO‐0.33 delivers a reversible capacity of 814 mA h g^−1^ after 600 cycles (Figure 3b). At 50 A g^−1^ (Figure S20, Supporting Information), IVO‐0.33 shows a stable cyclic performance for 10 000 cycles, delivering a reversible capacity of 124.9 mA h g^−1^. For rate performance (Figure 3c), the discharge capacity of IVO‐0.33 is 1126.1, 1190.5, 1230.1, 1216.2, 1156.5, and 1000.4 mA h g^−1^ at a current density of 0.1, 0.2, 0.5, 1, 2, and 5 A g^−1^, respectively, and a good recovery of capacity is achieved when the current density returned to 0.1 A g^−1^. The high‐rate performance and superior cycle stability of IVO‐0.33 is superior to most reported In‐based anode (Figure 3d; and Table S10, Supporting Information). To further testify the practical application of IVO‐0.33, a full cell (+) LiCoO_2_ || IVO‐0.33 (−) was fabricated in the CR2016 coin‐type cell. As depicted in Figure S22 (Supporting Information), the full cell features high coulombic efficiency (CE) of 81.1% and reach a capacity of up to 130.7 mA h g^−1^ over 1000 cycles with CE close to 100% at 1 A g^−1^, demonstrating the application of IVO electrode.

To further testify the universality of the strategy, we subsequently used a similar synthetic route to obtain solid solution (Sn,V)O_2_ (SVO, Figure S3, Supporting Information). As an alloying‐type element, Sn suffers huge volume expansion (≈300%) after lithiation, therefore, the capacity decays faster. From Figure S23a (Supporting Information), XRD pattern of SVO matches well with the crystallographic data of SnO_2_ (JCPDF#41−1445). Benefitting from the robust V−O frameworks, SVO displays stable cyclic performance compared with SnO_2_ (Figure S23b,c,). At 0.1 A g^−1^, SVO delivers a capacity of 962.4 mA h g^−1^ after 120 loops with ICE of 74.3%. To rational compare, Supporting Information the capacity of SnO_2_ decays rapidly to 771 mA h g^−1^ after merely 50 cycles (ICE: 61.0%). Besides, SVO displays better rate capability than SnO_2_ (Figure 3e). At 5 A g^−1^, SVO delivers a capacity of 700.1 mA h g^−1^. In comparison, SnO_2_ delivers a capacity of 183.2 mA h g^−1^. At relative higher current density of 2 A g^−1^, SVO delivers a stable capacity of 823.6 mA h g^−1^ after 600 cycles (Figure 3f). These results demonstrate the effectivity and universality of spreading alloying‐type elements in a robust intercalation‐type framework.

### 2.3 Kinetic Analysis

**Figure 4:**
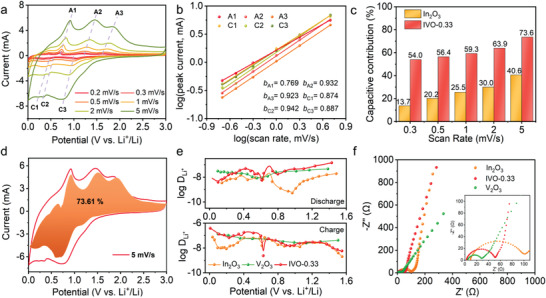
Potentiostatic cycling to understand the electrochemical behavior of IVO‐0.33 and In_2_O_3_. a) Cyclic voltammograms at varying sweep rates for IVO‐0.33 versus Li^+^/Li. b) Plot of log i versus log v of IVO‐0.33. c) Capacitive and diffusion‐controlled charge storage contribution with CV at 5 mV s^−1^. d) Capacity contribution of IVO‐0.33 and In_2_O_3_ from diffusion‐controlled process and capacitive at varying sweep rates. e) GITT curves showing the diffusion coefficients of IVO‐0.33, In_2_O_3_, and V_2_O_3_. f) The Nyquist plot of IVO‐0.33, In_2_O_3_, and V_2_O_3_.

The electrochemical kinetics of Li^+^ storage in IVO‐0.33 and In_2_O_3_ are further studied by multisweep rate cyclic voltammetry. CV curves of IVO‐0.33 (**Figure** **4**a) and In_2_O_3_ (Figure S24a, Supporting Information) with sweep rates (*v*) varying from 0.2 to 5 mV s^−1^ are obtained. The electrochemical behavior is investigated according to the Randles–Sevcik (R–S) equation: *i* = *aν^b^* or log (*i*) = *b*log (*ν*) + log *a*, where a and b‐value refer to adjustable parameters. The *b* = 1 indicates a Faradic contribution from charge transfer with the surface, while *b* = 0.5 means a diffusion‐controlled process. The *b*‐value could be determined by plotting log *i* versus log *ν*. From the linear fitting shown in Figure 4b; and Figure S24b (Supporting Information), *b*‐values of IVO‐0.33 from the slope of A1, A2, A3, C1, C2, and C3 are determined to be 0.77, 0.93, 0.92, 0.87, 0.94, and 0.89, respectively, indicating the reaction was capacitive‐dominating. To rational compare, b‐value from the slope of peak A1, A2, C1, and C2 are close to 0.5 in In_2_O_3_, indicating a Li diffusion‐controlled process. To quantify the capacitive contribution, the whole current (i) under a certain potential (V) is separated into diffusion‐controlled (proportional to the *v* ^0.5^) and capacitive contributions (linear relationship with *v*) based on the equation of *i* = *k*_1_ *v*+*k*_2_ *v* ^0.5^ (*k*_1_ and *k*_2_ were constant). From Figure 4c,d, the capacitive contribution for the total capacity at 0.3 mV s^−1^ is 54.0% and increase to 73.6% at 5 mV s^−1^ (displayed as orange area in Figure 4d). For In_2_O_3_, the capacitive contribution for the total capacity at 0.3 mV s^−1^ is 13.7% and increase to 40.6% at 5 mV s^−1^. Therefore, a pseudocapacitive charge storage mechanism could be achieved for IVO‐0.33 which enables high‐rate energy storage.

To measure the diffusion coefficient *D*_Li_^+^ of IVO‐0.33, In_2_O_3_ and V_2_O_3_, the galvanostatic intermittent titration technique (GITT) is performed (Figure 4e; and Figure S25a,b,). *D*_Li_^+^ is calculated by Fick's second law

(1) $$D_{{\mathrm{Li}}^{+}}=\frac{4}{\pi \tau }{\left(\frac{nV}{A}\right)}^{2}{\left(\frac{\Delta E_{S}}{\Delta E_{t}}\right)}^{2}$$

where *τ*, *n*, *V*, and *A* are relaxation time, mole of active material, molar volume of active material, and geometric area of electrode, respectively. *∆E*_s_ and *∆E*_t_ are potential changes occurred in steady‐state and current pulse, respectively. *D*_Li_^+^ of IVO‐0.33 at lithiation state varies from 3.45 × 10^−9^ to 1.45 × 10^−7^ cm^2^ s^−1^, and ranges from 2.35 × 10^−10^ to 3.91 × 10^−7^ cm^2^ s^−1^ at Li^+^ extraction state, which are higher than In_2_O_3_. Electrochemical impedance spectroscopy (EIS) further proves the smaller charge transfer resistance (*R*_ct_, determined by fitting the diameter of the semicircle in Nyquist plot) of IVO‐0.33 (49.1 Ω, fitted by Zview) than that of In_2_O_3_ (91.5 Ω, fitted by Zview), indicating that the introduction of V facilitates charge transfer (Figure 4f). Besides, the diffusion coefficient of Li^+^ can be estimated by the following formula

(2) $$D_{{\mathrm{Li}}^{+}}={\left(\frac{2RT}{\sqrt{2}\sigma ACn^{2}F^{2}}\right)}^{2}=\frac{2R^{2}T^{2}}{\sigma^{2}A^{2}C^{2}n^{4}F^{4}}$$

where *n*, *F*, *C*, *A*, *R*, and *T* are mole of charge transfer during lithiation/delithiation process, Faradic constant, concentration of Li^+^, the surface area of the anode, the gas constant, and room temperature, respectively. The *σ* is the Warburg coefficient which has the relationship with real part of impedance *Z*’ as follow formula: *Z’* = *R*_s_ + *R*_ct_ + *σω* ^−0.5^, where the *R*_s_ and *R*_ct_ are resistance of solution and charge‐transfer resistance, respectively. To investigate the Li^+^ diffusion behavior, the real part of impedance *Z*’ at low‐frequency region with the square root of angular frequency *ω* linearly is fit. From the above analysis, the diffusion coefficient is inverse with the value of *σ* ^2^, by which the diffusion coefficient of Li^+^ can be estimated. From the fitting results shown in Figure S26 (Supporting Information), the slope of the IVO‐0.33 is smaller than In_2_O_3_, inferring that IVO‐0.33 displayed fast reaction kinetics.

### 2.4 Li‐Storage Mechanisms and Discussions

**Figure 5:**
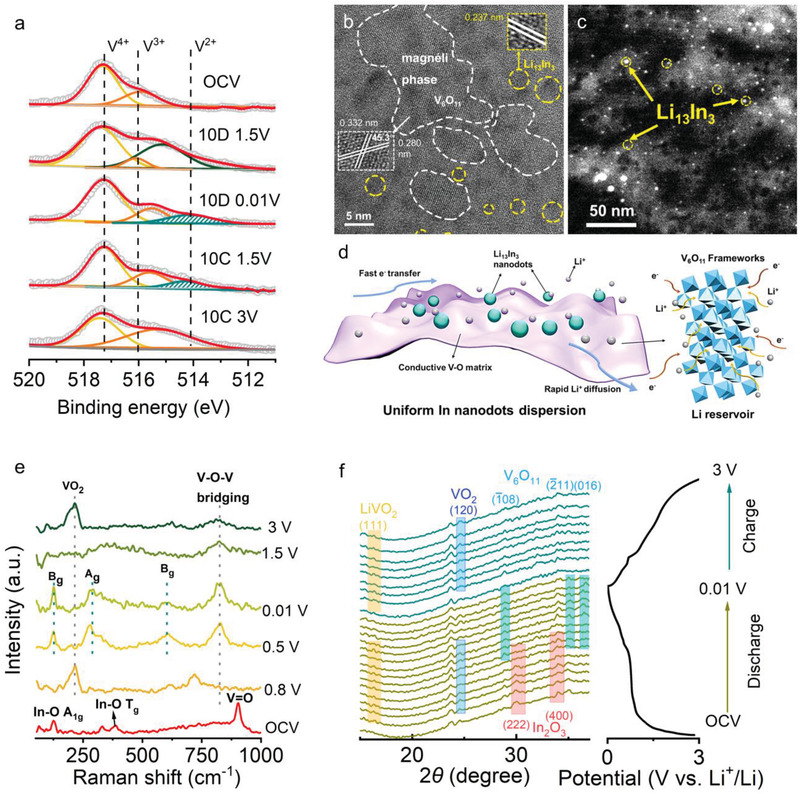
The investigation of lithium‐ion storage mechanism of IVO‐0.33. a) Deconvoluted V 2p spectra of IVO‐0.33 at various discharge/charge stages. b) Ex situ HRTEM of IVO‐0.33 for discharge to 0.01 V after 10 cycles, presenting the lattice distances of Li_13_In_3_ and V_6_O_11_. c) HADDF‐STEM image displayed uniform in situ formed Li_13_In_3_ nanodots dispersed in lithiated magnéli phase V_6_O_11_ matrix. d) Schematic illustration of uniform in situ formed Li_13_In_3_ nanodots dispersed in lithiated magnéli phase V_6_O_11_ matrix after fully lithiation. e) Ex situ Raman spectra of IVO‐0.33 at various discharge/charge stages. f) In situ XRD pattern of IVO‐0.33 in 1st cycle with charge–discharge profile.

To investigate the lithium storage mechanism of IVO‐0.33, in situ and ex situ characterizations are carried out. **Figure** **5**b; and Figure S27 (Supporting Information) displays the ex situ HRTEM images discharging to 0.01 V after 10 cycles. The interplanar distance of 0.237 nm could be attributed to the lattice plane of (440) of Li_4.33_In, indicating a thorough lithiation of indium (yellow circles in Figure 5b). The HRTEM images displays lattice fringes of 0.332, 0.322, and 0.280 nm, corresponding to (1$\bar{2}$0), ($\bar{1}16$), and (0$\bar{2}$6) plane of V_6_O_11_ (JCPDF #18‐1451), respectively. The angle between (1$\bar{2}$0) and ($0\bar{2}6$) plane is measured to be 45.3°, which is closed to theoretical value of 45.5° (white lines in Figure 5b). The measured angle between (1$\bar{2}$0) and ($\bar{1}16$) plane is 121.7°, which is similar with the theoretical value of 122.1° (Figure S27, Supporting Information). Corresponding Fourier fast transform (FFT, Figure S27, Supporting Information) image also displays ($\bar{1}16$) and (1$\bar{2}$0) of V_6_O_11_ after fully lithiation. From HADDF‐STEM image (Figure 5c), the Li−In alloy is presented as bright nanodots (yellow circles in Figure 5c) and distributed uniformly among Li−V−O matrix after lithiation. It is well known that V_6_O_11_ is a magnéli phase contributing a large lithium capacitive storage. Such a high conductive framework relieves the volume expansion of alloying‐type In nanodots (Figure 5d). The Li‐extraction of Li−In alloy is also investigated (Figure S28, Supporting Information). From Figure S28 (Supporting Information), lattice distances of 0.330 and 0.294 nm can be indexed to the lattice fringe (1$\bar{2}$0) and (108) for V_6_O_11_, proving the structure stability of V_6_O_11_ after repeated cycling. Besides, an interplanar distance of 0.272 nm is indexed to (101) of In, and some particles display interplanar distance of 0.289 nm, which could be indexed to (104) of R$\bar{3}$c In_2_O_3_, showing enhanced electrochemical reversibility. SAED image (Figure S29b, Supporting Information) reveals opaque diffraction rings, indicating an amorphous situation of IVO‐0.33 after fully Li‐extraction. After charging to 3 V at 50th cycle, IVO‐0.33 still displayed as isolated particles in Figure S31a (Supporting Information), while In_2_O_3_ exhibited an agglomeration in Figure S31b (Supporting Information). Therefore, In nanodots are uniformly dispersed in V−O matrix uniformly after many cycles.

To further investigate lithium storage mechanism, ex situ XPS (Figure 5a), ex situ Raman (Figure 5e), and in situ XRD (Figure 5f) at various cutoff potentials based on charge/discharge curves are performed. Ex situ deconvoluted V 2*p* spectra at various cutoff potentials are achieved to investigate the change of valence state. Briefly, in initial stage the deconvoluted V 2*p* signals at 517.2 and 516.1 eV reveals the existence of V^IV^ and V^III^, respectively. When discharged to 1.5 V, a peak at 515.5 eV could be deconvoluted, indicating a transformation from V^III^ to a lower valence state, which is corresponding to the lithiation of V−O framework in (In,V)_2_O_3_. Further lithiation to 0.01 V generates a peak at 514.2 eV, indicating the existence of V^II^. The lithium extraction at 1.5 V increases the percentage of V^III^ component, implying a recovery of V^III^ from V^II^. After charged to 3 V, the peak of V^II^ disappears and the valence states return to V^III^ and V^IV^, revealing a reversible lithium storage of V−O frameworks. No V^0^ signals (512.4 eV) appears during the whole lithiation and Li‐extraction process,^[^ ^47^ ^]^ revealing that the robust V−O frameworks stored Li^+^ under a reversible intercalation/deintercalation process without breaking of V−O bond rather than conversion mechanism.

Ex situ Raman further proves the phase transformation. After discharged to 0.8 V, a new Raman peak at 212 cm^−1^ appears, which can be attributed to bending vibration of V−O. After lithiated to 0.5 V, Raman peaks of 123, 294, and 827 cm^−1^ are observed, corresponding to skeleton bending vibration of V−O−V, V−O bending and bridging asymmetric stretching V−O−V, respectively.^[^ ^28^, ^48^ ^]^ The results proves the generating of VO*_x_* after lithiation. After charging to 3 V, the vibration mode of V−O−V and bending of V−O can be maintained, implying the stability of V−O frameworks during Li insertion and extraction. From the in situ XRD pattern, the discharge process causes the diffraction peaks of In_2_O_3_ (JCPDF#06−0416) faded away accompanied by intensified peaks of VO_2_ (JCPDF#25−1003). After discharging to 0.8 V, a phase reconstruction occurs, generating new magnéli phase V_6_O_11_ (JCPDF#18−1451) with the decrease intensity of VO_2_.^[^ ^49^ ^]^ The lattice fringe and electron diffraction rings of cubic LiVO_2_ (JCPDF#36−0041) are observed in HRTEM and SEAD at a stage of discharging to 1.5 and 0.8 V after 10 cycles (Figure S30a,b, Supporting Information). The formation of cubic LiVO_2_ is thermodynamic‐favorable in Li_2_O–V_2_O_3_ system.^[^ ^50^, ^51^ ^]^

From the above characterizations, we assume that the lithium storage mechanism of IVO‐0.33 can be written as follows

(3) $$\begin{array}{ccc} {\mathrm{In}}_{1.33}V_{0.28}^{\mathrm{III}}V_{0.28}^{\mathrm{IV}}O_{3}+4e^{-}+4\;Li^{+} & = & 1.33\;\mathrm{In}+0.14V_{2}O_{3}\\ & & +\;0.28\;VO_{2}+2Li_{2}O \end{array}$$

(4) $$3\;\mathrm{In}+13\;Li^{+}+13e^{-}=In_{3}{\mathrm{Li}}_{13}$$

(5) $$V_{2}O_{3}+Li_{2}O=2\mathrm{LiV}O_{2}$$

(6) $$4VO_{2}+V_{2}O_{3}=V_{6}O_{11}$$

(7) $$x{\mathrm{Li}}^{+}+V_{6}O_{11}+xe^{-}=Li_{x}V_{6}O_{11}$$

During the first discharge, the (In,V)_2_O_3_ solid solution undergoes a conversion reaction at a cutoff potential of 0.8 V, forming In nanodots dispersed in V_2_O_3_ and VO_2_. Following lithiation promotes the generation of magnéli phase V_6_O_11_ with the consumption of V_2_O_3_ and VO_2_, which is also supported by thermodynamics in V_2_O_3_–VO_2_ system.^[^ ^49^, ^52^ ^]^ At a potential range of 0–0.8 V, alloying‐reaction products Li_13_In_3_ nanodots trapped in lithiated V_6_O_11_ networks. Such robust Li−V−O networks facilitates the diffusion of Li^+^ and hindered the aggregation of Li–In alloy. After Li‐extraction, most Li_13_In_3_ nanoparticles are converted to In nanoparticles while In_2_O_3_ could be partially recovered and Li*_x_* V_6_O_11_ could be partially decomposed to V_2_O_3_ (which could further react with Li_2_O to form LiVO_2_) and VO_2_, showing better reaction reversibility.

## 3 Conclusion

In summary, we proposed a universal model of high capacity and high‐rate anodes which contains well‐spread alloying‐type element in the robust intercalation‐type framework. The alloying‐type element provides high capacity, while its agglomeration was alleviated by the intercalation‐type framework, resulting in good cyclic stability and rate capability. Benefited from above merits, (In,V)_2_O_3_ solid solution delivered high gravimetric capacity of 1364 mA h g^−1^ at 1 A g^−1^ with a safe average discharge potential of about 0.6 V, as well as an excellent cycling stability at large current densities up to 50 A g^−1^. It was verified that (In,V)_2_O_3_ solid solution was transformed to Li_13_In_3_ nanodots dispersed in stable Li*_x_* V_6_O_11_ frameworks after fully lithiation. Pseudocapacitive V_6_O_11_ frameworks offered extra Li‐storage sites, accelerated the Li^+^ diffusion and hindered the aggregation of indium particles. After fully extraction of Li^+^, In could be partially reoxidized to hexagonal In_2_O_3_, indicating an increment of the reversibility of electrode reaction. Confining alloying motifs in intercalative frameworks could be extended effectively to other alloying‐type anodes including (Sn,V)O_2_, (Sn,Ti)O_2_, and binary oxide, providing a universal way to simultaneously achieve high capacity and high‐rate performance.

## 4 Experimental Section

### Material Synthesis

The (In,V)_2_O_3_ solid solution was synthesized by two methods: solvothermal method and a solid‐state reaction. To obtain IVO‐*x* (*x* was defined as vanadium atomic percentage, *x* = 0, 0.25, 0.33, 0.5, 0.66, and 0.83), a typical solvothermal method was adopted. For a typical synthesis of IVO‐0.33, 2 mmol In(Ac)_3_ and 0.5 mmol V_2_O_5_ were dispersed in 30 mL EtOH solvent, then stirring for 1 h to obtain an orange suspension. Afterward, the suspension was transferred to a 50 mL Teflon‐lined autoclave and reacted at 180 °C for 24 h. After cooled down, the gray precipitate was collected by centrifugation and washed by ethanol and water several times, then dried at 80 °C for 12 h. The (In,V)_2_O_3_ nanoparticles were achieved by annealing the precipitate in Ar atmosphere at 600 °C for 2 h. IVO‐0.21 was synthesized by a solid‐state reaction to further investigate the physical properties. Briefly, the In_2_O_3_ and equimolar amounts of V_2_O_3_ with a nominal composition of In_0.79_V_0.21_O_3_ were mixed followed by grinding. The mixed powders were pressed into a ceramic plate, followed by calcinating at 600 °C for 24 h in Ar atmosphere. The gray (In,V)_2_O_3_ powder can be obtained after calcination. Contrast In_2_O_3_ was obtained by calcinating In_2_O_3_ stock at 600 °C for 24 h in Ar atmosphere. The synthetic process of (Sn,V)O_2_ solid solution (SVO) was similar with (In,V)_2_O_3_ solid solution except for using 2 mmol Sn(Ac)_2_ and 0.5 mmol V_2_O_5_. SnO_2_ was synthesized in the same way except for the addition of V_2_O_5_.

### Sample Characterization

The PXRD pattern of the product was collected by D2 Bruker X‐ray diffractometer with Cu K*α* radiation (*λ* = 1.5418 Å) at a scan rate of 2° min^−1^. High resolution PXRD pattern were collected on PANalytical Empyrean X‐ray diffractometer with Cu K*α* line focused radiation at 40 kV and 40 mA from 2*θ* = 5° up to 120° with 0.02° increment by Bragg–Brentano. The structure refinements were carried out by Le bail and Rietveld methods using the Jana program. The SEM was collected by the Hitachi S‐4800 electron microscope, running at 5 kV. XPS were collected by Axis Ultra spectrometer (Kratos Analytical, Al K*α* radiation). UPS and XPS‐VB were collected by Axis Ultra spectrometer (Kratos Analytical, He I emission at 21.2 eV). Raman spectra were collected on a Thermal Fisher Micro Raman imaging spectrometer (DXRxi) using a 532 nm laser. TEM, SAED, and (HAADF‐STEM) images were collected by JEM‐2100F microscope (JOEL). Inductively coupled plasma atomic emission spectrometry (ICP‐AES, Prodigy 7) was carried out to analyze the elemental composition. UV–vis spectra were obtained through Shimadzu UV3600Plus UV–VIS–NIR spectroscopy. Carrier concentration and mobility were measured based on Hall effect through Physical Properties Measurement System (PPMS). The relationship between magnetic induction intensity (*B*) and carrier concentration (*n*) can be express as *n* = *IB*/*V*_H_ *ed*, where *d* was the thickness of ceramic plates, *e* was the charge of a single electron, *I* was current, and *V*_H_ was the Hall voltage. The carrier mobility (*μ*) could be deduced from the equation of *μ = σ/ne*, where *σ* was the conductivity. By linear fitting the plot *V*_H_/*I* versus *B* (equal to resistivity *R* vs *B*), the carrier concentration and the carrier mobility could be calculated from the slope (denoted as *R*_H_).

### Theoretical Calculation

The Vienna Ab‐initio Simulation Package (VASP) was applied for DFT calculation. Ultrasoft (US) pseudopotentials and Perdew–Burke–Ernzerhof (PBE) parameterization of the generalized gradient approximation (GGA) were adopted for the core‐valence electron interaction and exchange‐correlation functional. The cutoff energy was set as 450 eV. The total energy convergence was less than 10^−6^ eV per atom. The force‐on‐atom was converged below a threshold of 0.02. For In_2_O_3_ cell, the Monkhorst‐Pack K‐point grid was set as 2 × 2 × 2 for geometrical optimization. For (In,V)_2_O_3_, a In atom was substituted by V. The GGA + *U* method was used in the density of states for V‐doped In_2_O_3_, where *U* the Hubbard parameter, and *J* the exchange parameter was 3.25 and 0 eV, respectively.

### Electrochemical Measurements

The working electrode was fabricated by mixing active material, acetylene black, and polyvinylidene difluoride (PVDF) with a weight ratio of 8:1:1 thoroughly in *N*‐methyl‐pyrrolidone (NMP) to form a slurry. Afterward, the slurry was coated on a Cu foil, and dried in 80 °C for 12 h. The electrode was cut into circular disks with a diameter of 14 mm. The mass loading of the anode was 1.2 mg cm^−2^. Electrochemical measurements were carried out by assembling the CR2016 coin‐type cell in an argon‐filled glovebox. In a coin‐type cell, glass fiber (Whatman) was used as separator and lithium plate was used as counter electrode. 1 m LiPF_6_ in a solution of ethyl carbonate (EC) and diethyl carbonate (DEC) (w/w = 50:50) added with 10.0% fluoroethylene carbonate (FEC) and 1.0% vinylene carbonate (VC) was used as electrolyte. Cyclic voltammetry (CV) and electrochemical impedance spectroscopy (EIS) was carried out on a CHI660 electrochemical workstation. Galvanostatic cycling performance and rate capability test were carried out on LAND Battery Test System in a voltage‐range of 0.01–3 V.

The full cell (+) LiCoO_2_ || IVO (−) was fabricated in the CR2016 coin‐type cell. LiCoO_2_ (180 mA h g^−1^, maximum voltage 4.5 V) and IVO‐0.33 were used as cathode and anode, respectively. The N/P ratio was ≈1. 1 m LiPF_6_ in EC/DEC (w/w = 50:50) added with 10.0% FEC and 1.0% VC was used as electrolyte. The mass loading of LiCoO_2_ was 18 mg cm^−2^. The working voltage was 2.5–4.2 V.

### Theoretical Capacity Calculation

The electrode reactions in In_2_O_3_ anode are summarized as following

(8) $$In_{2}O_{3}+6e^{-}+6Li^{+}=2\;\mathrm{In}+3Li_{2}O$$

(9) $$\mathrm{In}+4.33Li^{+}+4.33e^{-}=\mathrm{InL}i_{4.33}$$

The theoretical capacity is calculated according to equation: *C* = *nF*/3.6*M*, where *n* is the number of transferred electrons, *F* is Faraday constant (96 485 C mol^−1^), and *M* is the molar mass of In_2_O_3_ (277 g mol^−1^).

By *C* = *nF*/3.6*M*, reaction 1) offers a theoretical capacity of 579.0 mA h g^−1^, while reaction 2) is completely reversible and provides a capacity of 418.0 mA h g^−1^ on the basis of In_2_O_3_ start material. The overall capacity of In_2_O_3_ is 1415 mA h g^−1^ (579 + 2*418).

## Conflict of Interest

The authors declare no conflict of interest.

## Supporting information

Supporting Information

## Data Availability

Research data are not shared.
