# Supplementary material for: Alloying Motif Confined in Intercalative Frameworks toward Rapid Li‐Ion Storage
Source: Adv Sci (Weinh). 2022 Jun 17;9(23):2202026. doi: 10.1002/advs.202202026 (PMC9376843; doi:10.1002/advs.202202026)
Supplement: Supplementary file 1 — Supporting Information [file ADVS-9-2202026-s001.pdf]

Supporting Information

Alloying motif confined in intercalative frameworks towards rapid Li-ion storage

Xueyu Lin, [a] *Chenlong Dong,* [a] *Siwei Zhao* [a], *Baixin Peng,* [b] *Ce Zhou ,* [a] *Ruiqi Wang* [a,] * *and Fuqiang Huang*[a,b]*

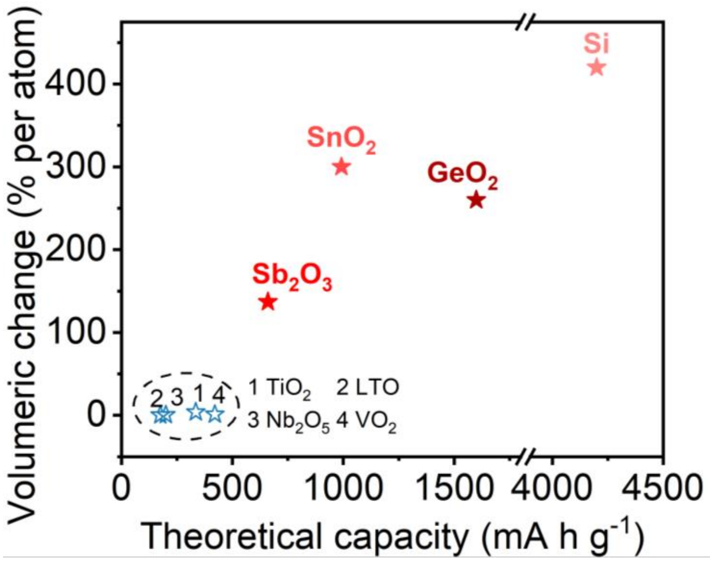

Figure S1. Summary of volumetric change for typical intercalation-type, alloying-type and conversion-alloying-type anode.[1, 2] Alloying-type or conversion-alloying-type anode (red stars in the figure) delivers high theoretical capacity but suffer severe volumetric change, while Intercalation ones experiences negligible volumetric change but delivers fewer capacity.

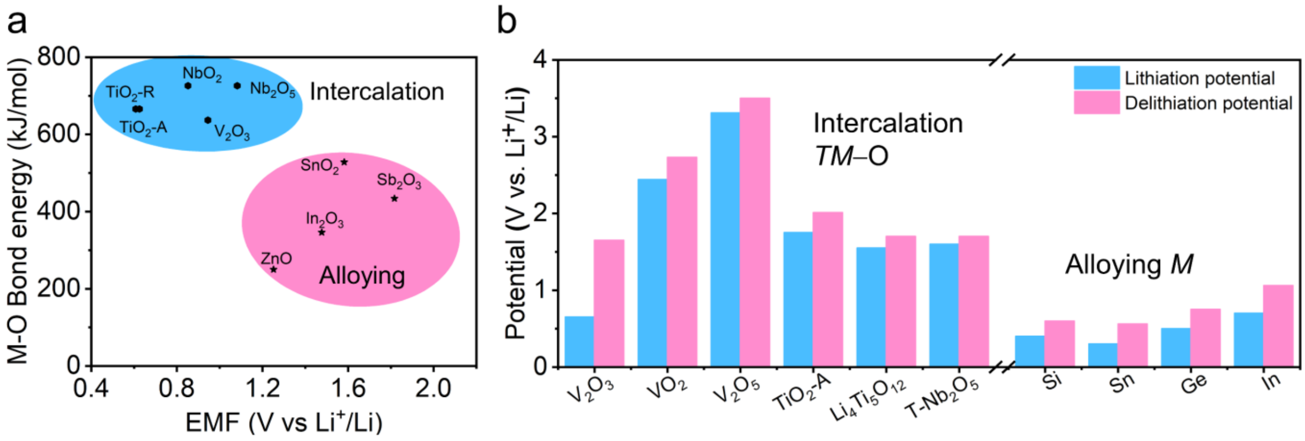

Figure S2. **a)** Summary of electromotive force (EMF) calculated from Gibbs free energy of formation for binary metal oxide and bond energies among typical intercalation-type, conversion-type and conversion-alloying-type

anodes.[3, 4] Higher *M*–O bond energy ensure a stable crystal structure during lithiation, leading to a low EMF. Lower *M*–O bond energy enable the *M*–O bonding to be easily broken after lithiation, resulting in a conversion from metal oxide to *M*/Li_2O matrix, displaying a high EMF. **b)** Comparison of lithiation and delithiation potential among typical intercalation-type and alloying-type anode base on initial distinct charge/discharge plateau.**[5-26]**

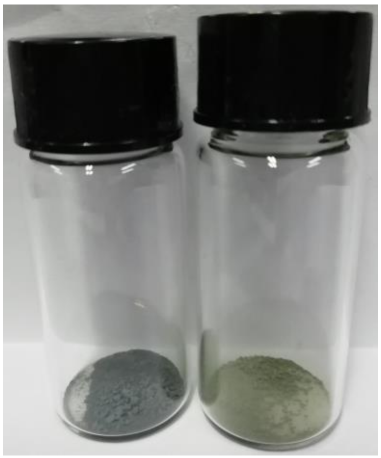

Figure S3. Photograph of blue grey IVO-0.33 (left) and green SVO (right).

Table S1. ICP-AES results of $(In,V)_2O_3$ synthesized with different In/V atomic ratio.

**Samples**	**Metal compositions (mol%)**		**In/V ratio**	**Chemical formula**
	**In**	**V**		
IVO-0.25	0.053	0.015	0.560	$In_{1.56}V_{0.44}O_3$
IVO-0.33	0.040	0.017	2.356	$In_{1.40}V_{0.60}O_3$
IVO-0.50	0.044	0.036	1.225	$In_{1.10}V_{0.90}O_3$
IVO-0.66	0.023	0.042	0.560	$In_{0.72}V_{1.28}O_3$
IVO-0.21	0.052	0.014	3.768	$In_{1.58}V_{0.42}O_3$

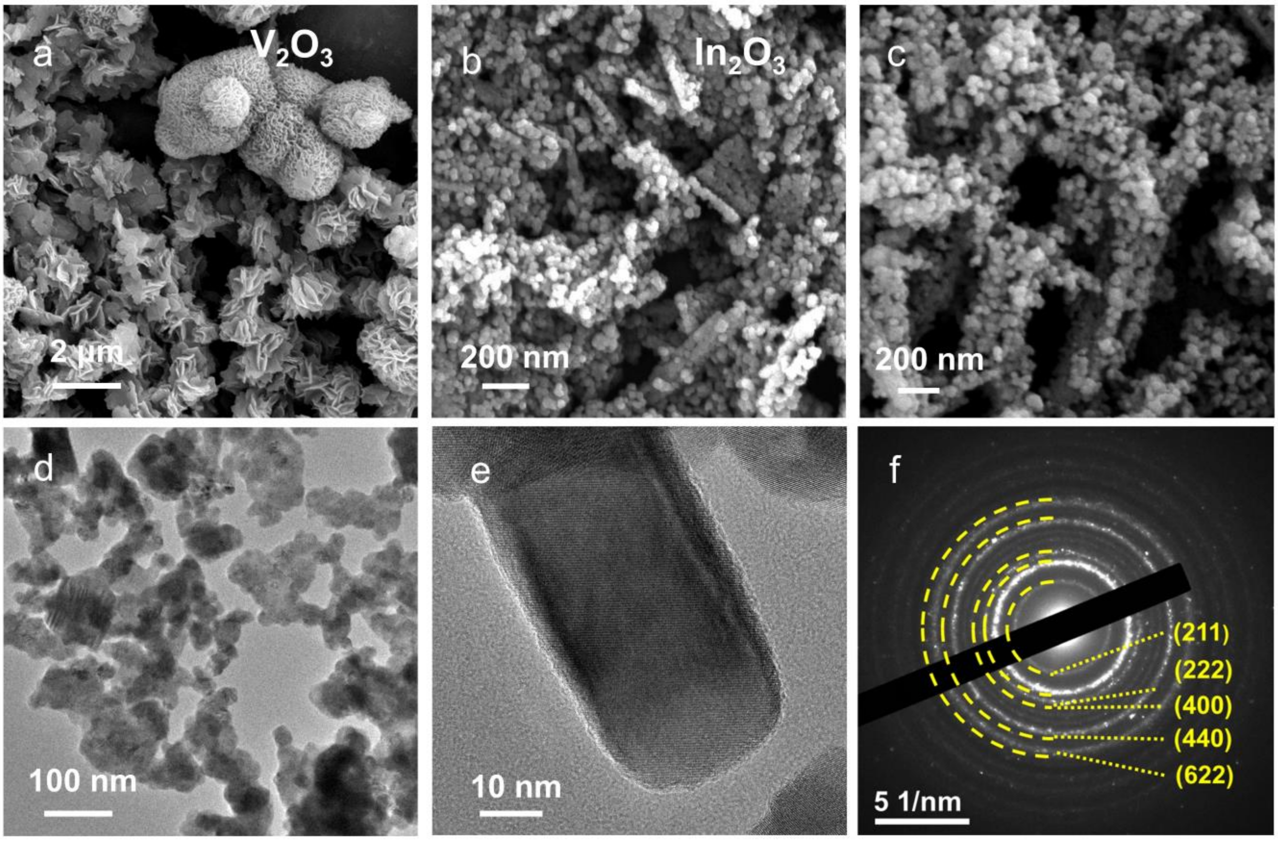

Figure S4. Morphology of V_2O_3, In_2O_3 and IVO-0.33. SEM image of **a)** V_2O_3, **b)** In_2O_3 and. **c)** IVO-0.33, **d,e)** TEM images and **f)** corresponding SAED pattern of IVO-0.33, showing a good crystalline and no inter planar of VO_x can be detected.

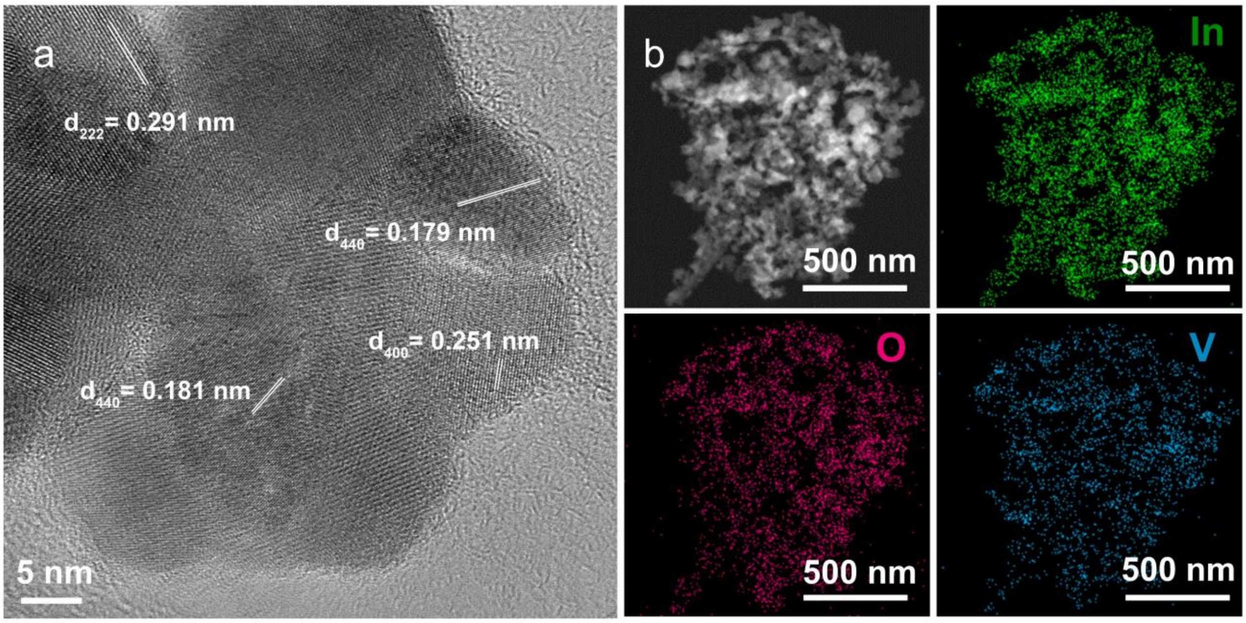

Figure S5. Microstructure of IVO-0.33. **a)** HRTEM images of IVO-0.33 HAADF-STEM image and **b)** elemental

mapping of IVO-0.33.

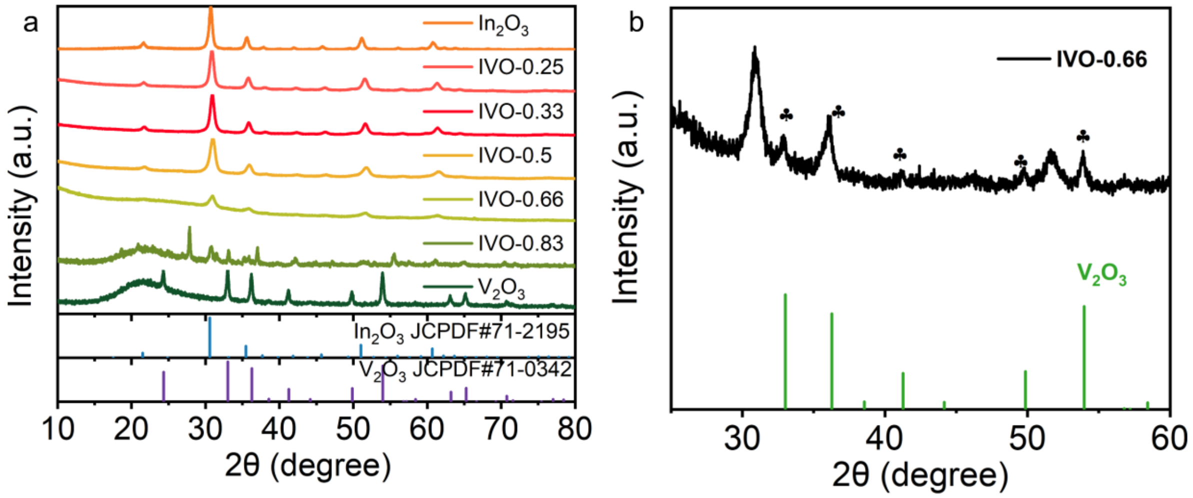

Figure S6. **a)** XRD pattern of IVO-*x* (*x* = 0.25, 0.33, 0.5, 0.66 and 0.83), In_2O_3 and V_2O_3. **b)** XRD pattern of IVO-0.66.

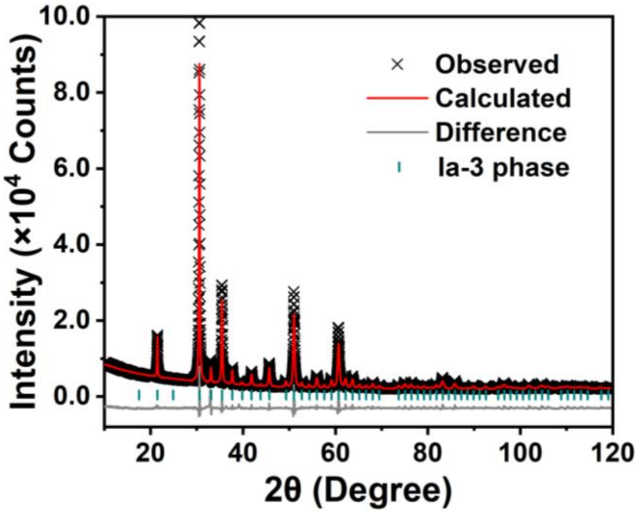

Figure S7. Rietveld refinement plot of synthesized In_2O_3 nanoparticles.

Table S2. Results of Rietveld analysis of synthesized $\mathbf{In_2O_3}$ nanoparticles shown in **Figure S7**.

$\mathbf{In_2O_3}$, Space group: **Ia-3**<br>a=b=c= 10.1120 Å<br>X-ray pattern: R_{wp}= 5.35 %					
Atom type	***WyckSymb***	***x***	***y***	***z***	***Occupancy***
In	8b	0.2500	0.2500	0.2500	1
In	24d	0.4682	0.0000	0.2500	1
O	48e	0.3860	0.1536	0.3849	1

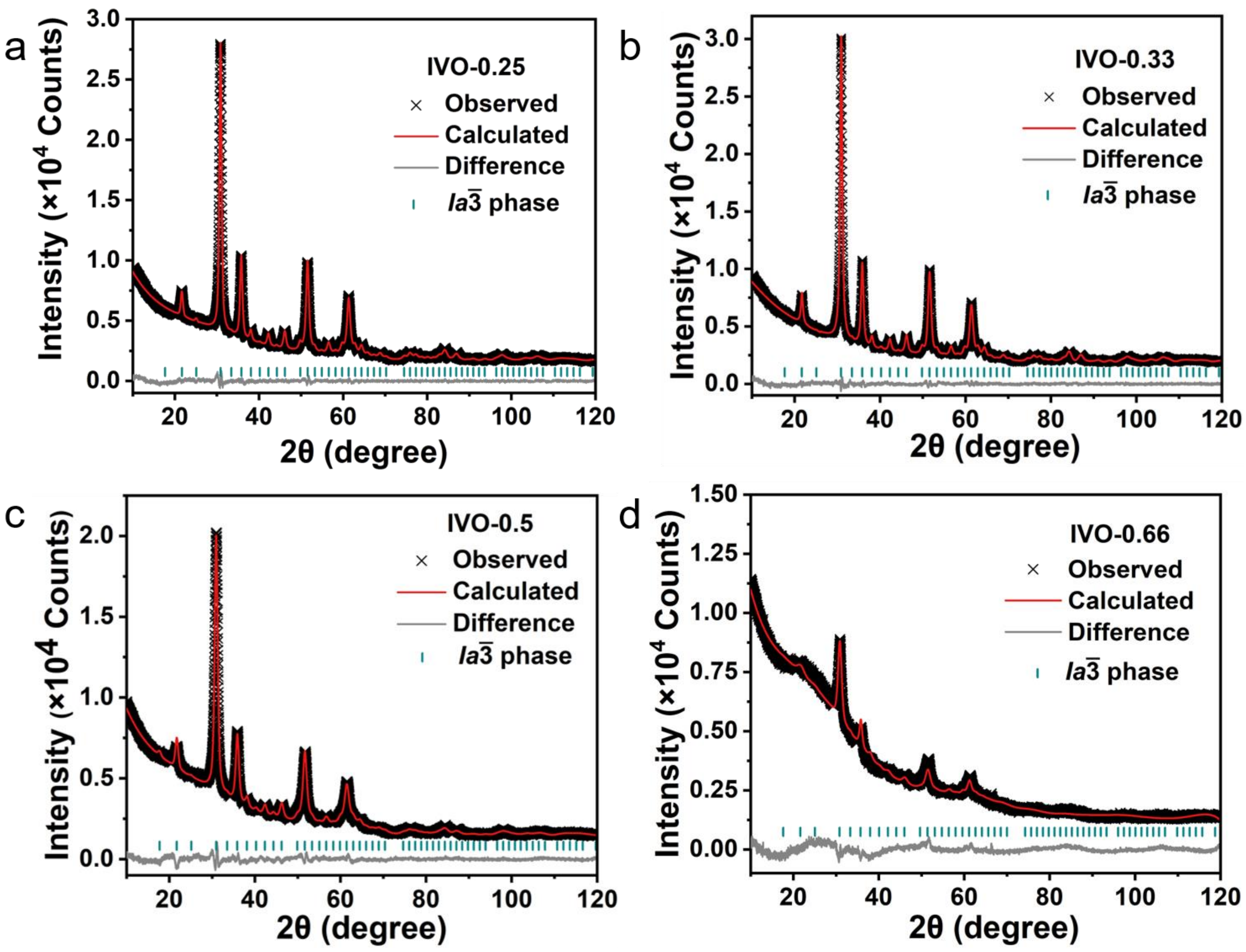

Figure S8. Rietveld refinement plots of $(In,V)_2O_3$ solid solution: **a)** IVO-0.25, **b)** IVO-0.33, **c)** IVO-0.5 and **d)** IVO-0.66.

Table S3. Results of Rietveld analysis of IVO-0.25 shown in **Figure S8a**.

Solid solution $\mathbf{In_{1.557}V_{0.443}O_3}$, Space group: **Ia-3**<br>a=b=c= 10.0177 Å<br>X-ray pattern: R_{wp}= 2.45 %					
Atom type	***WyckSymb***	***x***	***y***	***z***	***Occupancy***
In	8b	0.2500	0.2500	0.2500	1
V	8b	0.2500	0.2500	0.2500	0
In	24d	0.4668	0.0000	0.2500	0.86
V	24d	0.4668	0.0000	0.2500	0.14
O	48e	0.3905	0.1529	0.3832	1

Table S4. Results of Rietveld analysis of IVO-0.33 shown in **Figure S8b**.

Solid solution $\mathbf{In_{1.405}V_{0.595}O_3}$, Space group: **Ia-3**<br>a=b=c= 10.0157 Å<br>X-ray pattern: R_{wp}= 2.50 %					
Atom type	***WyckSymb***	***x***	***y***	***z***	***Occupancy***
In	8b	0.2500	0.2500	0.2500	0.7
V	8b	0.2500	0.2500	0.2500	0.3
In	24d	0.4695	0.0000	0.2500	0.81
V	24d	0.4695	0.0000	0.2500	0.19
O	48e	0.3975	0.1562	0.3896	1

Table S5. Results of Rietveld analysis of IVO-0.5 shown in **Figure S8c**.

Solid solution $\mathbf{In_{1.101}V_{0.899}O_3}$, Space group: **Ia-3**<br>a=b=c= 9.9936 Å<br>X-ray pattern: R_{wp}= 2.90 %					
Atom type	***WyckSymb***	***x***	***y***	***z***	***Occupancy***
In	8b	0.2500	0.2500	0.2500	1
V	8b	0.2500	0.2500	0.2500	0
In	24d	0.4695	0.0000	0.2500	0.74
V	24d	0.4695	0.0000	0.2500	0.26
O	48e	0.3905	0.1529	0.3832	1

Table S6. Results of Rietveld analysis of IVO-0.66 shown in **Figure S8d**.

Solid solution $\mathbf{In_{0.718}V_{1.282}O_3}$, Space group: **Ia-3**<br>a=b=c= 10.0484 Å<br>X-ray pattern: R_{wp}= 3.78 %					
Atom type	***WyckSymb***	***x***	***y***	***z***	***Occupancy***
In	8b	0.2500	0.2500	0.2500	0.18
V	8b	0.2500	0.2500	0.2500	0.82
In	24d	0.4668	0.0000	0.2500	0.08
V	24d	0.4668	0.0000	0.2500	0.92
O	48e	0.3905	0.1529	0.3832	1

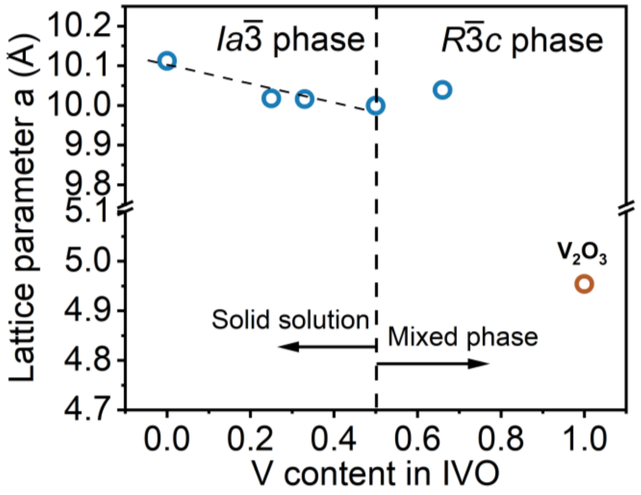

Figure S9. Refined lattice parameters of IVO-*x* (*x* = 0.25, 0.33, 0.5, 0.66 and 0.83), In_2O_3 and V_2O_3.

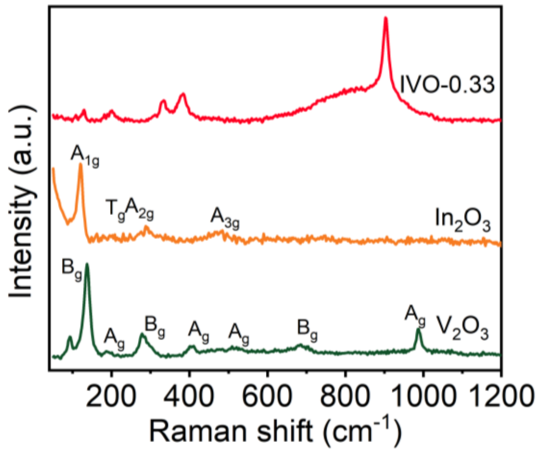

Figure S10. Raman spectra of IVO-0.33, In_2O_3 and V_2O_3.

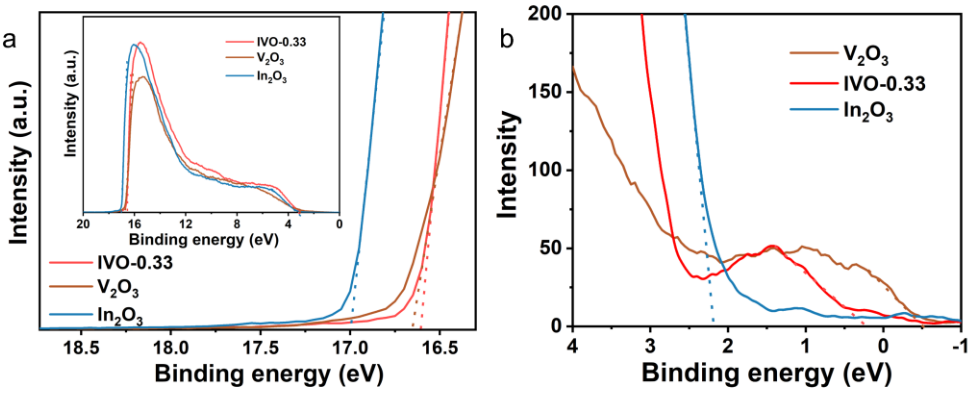

Figure S11. **a)** UPS results and cut-off edge at high binding energy of IVO-0.33, V_2O_3 and In_2O_3. **b)** XPS-VB spectra of IVO-0.33, In_2O_3 and V_2O_3.

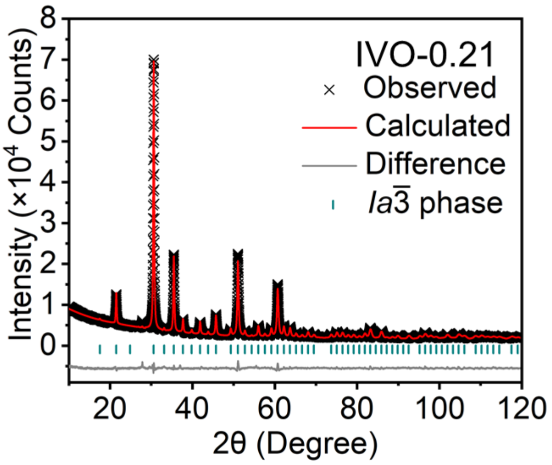

Figure S12. Rietveld refinement plots of IVO-0.21.

Table S7. Results of Rietveld refinement of IVO-0.21 shown in **Figure 12**.

Solid solution $\mathbf{In_{1.581}V_{0.419}O_3}$, Space group: **Ia-3**<br>a=b=c= 10.1131 Å<br>X-ray pattern: R_{wp}= 2.50 %					
Atom type	***WyckSymb***	***x***	***y***	***z***	***Occupancy***
In	8b	0.2500	0.2500	0.2500	0.81
V	8b	0.2500	0.2500	0.2500	0.19
In	24d	0.4659	0.0000	0.2500	0.78
V	24d	0.4659	0.0000	0.2500	0.22
O	48e	0.3950	0.1589	0.3809	1

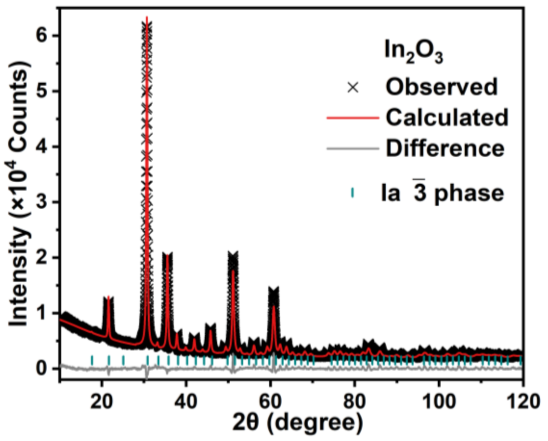

Figure S13. Rietveld refinement plot of contrast-In_2O_3.

Table S8. Results of Rietveld refinement of contrast-In_2O_3 shown in **Figure S13**.

In_2O_3, Space group: Ia-3<br>a=b=c= 10.1182 Å<br>X-ray pattern: R_{wp}= 4.89 %					
Atom type	***WyckSymb***	***x***	***y***	***z***	***Occupancy***
In	8b	0.2500	0.2500	0.2500	1
In	24d	0.4668	0.0000	0.2500	1
O	48e	0.3905	0.1529	0.3832	1

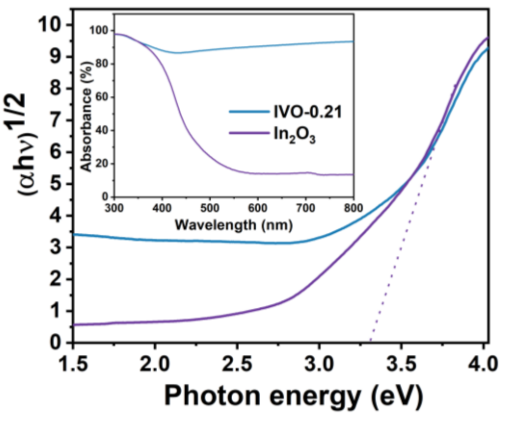

Figure S14. Absorption spectra of corresponding $(ahv)^{1/2}$ *vs*. photon energy plot for IVO-0.21 and In_2O_3. IVO-0.21 showed stronger absorbance in the visible region compared with In_2O_3, indicating the incorporation of V 3*d* states near the band edges.

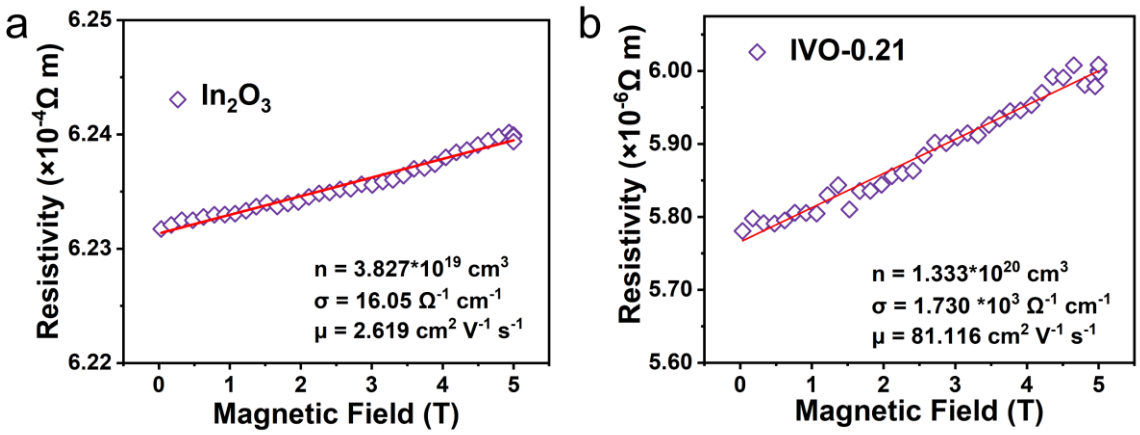

Figure S15. Plot of resistivity versus magnetic field for **a)** In_2O_3 and **b)** IVO-0.21.

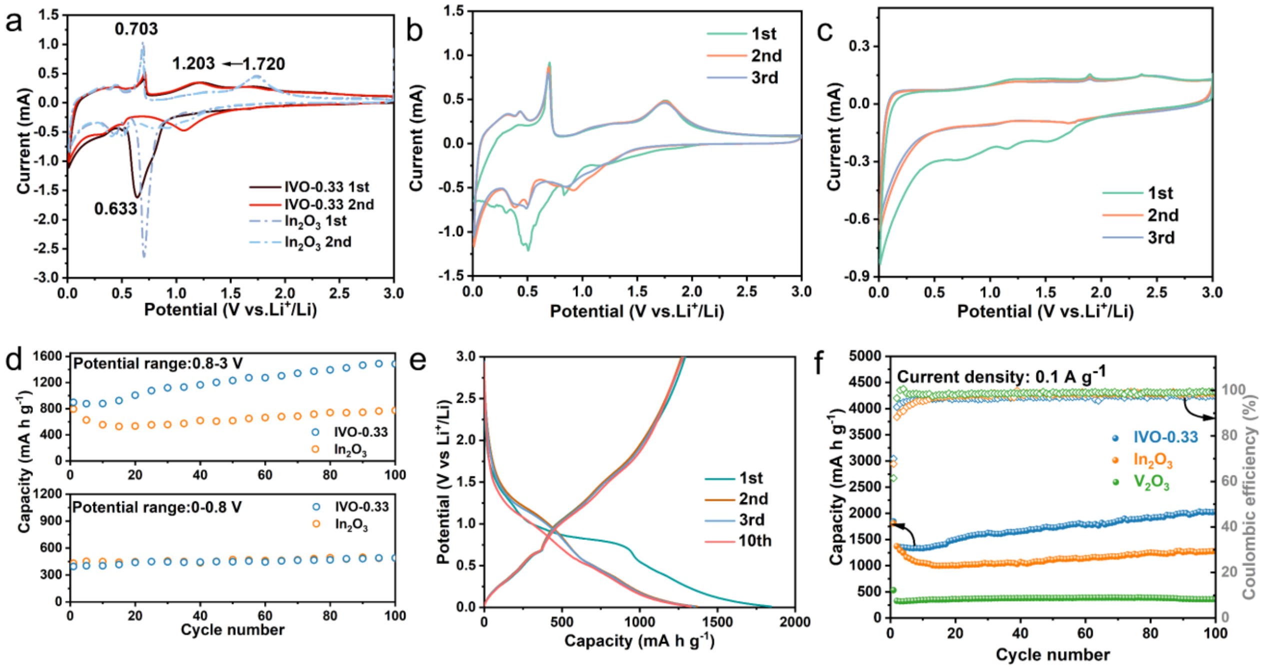

Figure S16. CV plots of **a)** IVO-0.33, **b)** In_2O_3 and **c)** V_2O_3. **d)** Capacity contribution at a potential range of 0-0.8 V and 0.8-3 V. **e)** Charge-discharge curves of IVO-0.33 at a current density of 0.1 A g^{-1}. **f)** Cycling performance of IVO-0.33 at a current density of 0.1 A g^{-1}.

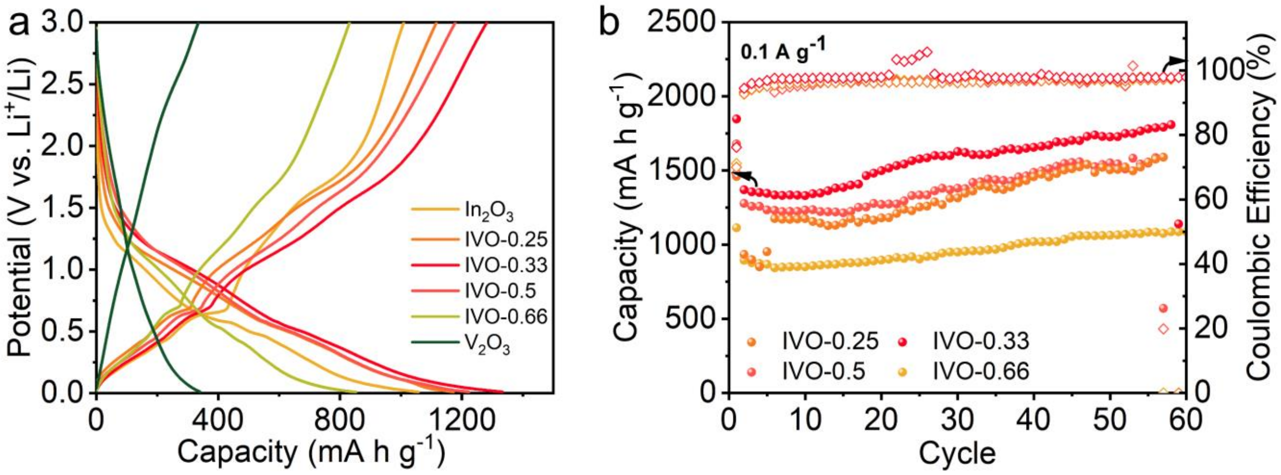

Figure S17. a) Charge-discharge curves of $(In,V)_2O_3$ solid solution with different In/V ratio at a current density of 0.1 A g^{-1}. **b)** Cycling performance of $(In,V)_2O_3$ solid solution with different In/V ratio at 0.1 A g^{-1}.

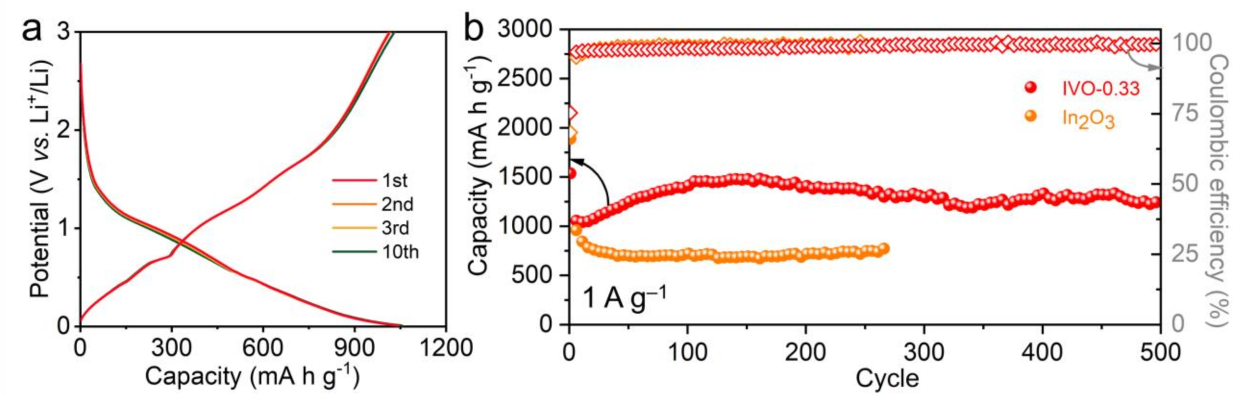

Figure S18. **a)** Charge and discharge voltage profiles of IVO-0.33 at 1 A g^{-1}. **b)** Cycling performance of IVO-0.33 at 1 A g^{-1}.

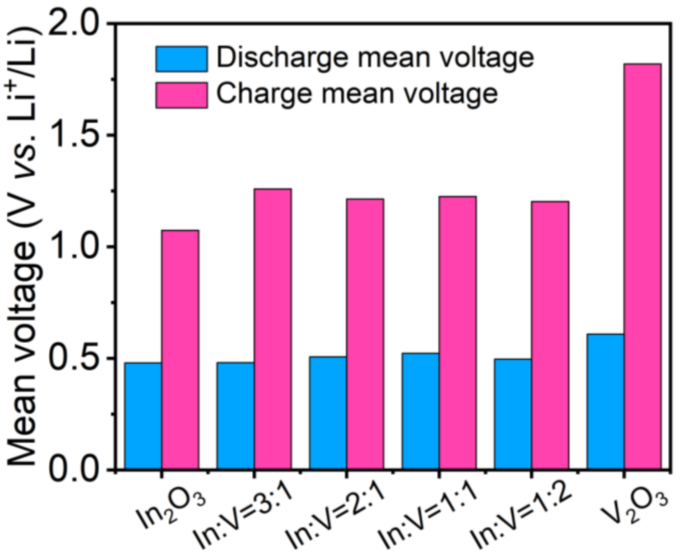

Figure S19. Comparison of discharge/charge mean voltage of IVO electrodes with different In/V ratios.

Table S9. Comparison of capacity, charge plateau and ICE of IVO electrodes with different In/V ratios.

Sample	In/V ratio	Capacity (mA h g^{-1})	Charge plateau (V vs Li$^+$/Li)	ICE(%)
In_2O_3	-	1109.2	1.073	67.74
IVO-0.25	In:V=3:1	1504.5	1.259	68.19
IVO-0.33	In:V=2:1	1726.9	1.214	73.90
IVO-0.5	In:V=1:1	1550.3	1.225	74.60
IVO-0.66	In:V=1:2	1062.4	1.202	76.21

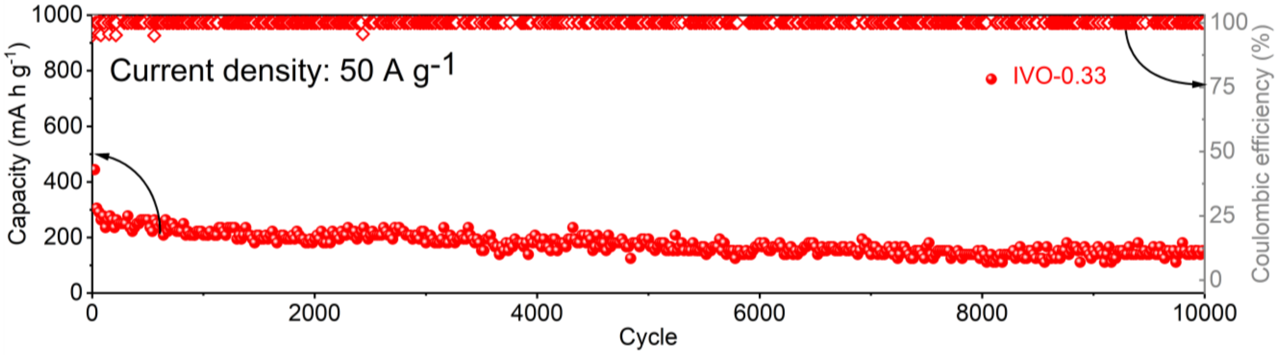

Figure S20. The capacity of IVO-0.33 at 50 A g^{-1} in long-term cycling (First 10 cycles: 0.1 A g^{-1}).

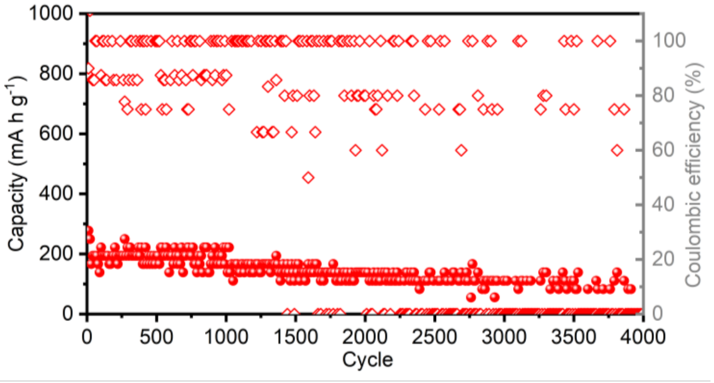

Figure S21. The capacity of IVO-0.33 at 100 A g^{-1} in long-term cycling (First 10 cycles: 0.1 A g^{-1}).

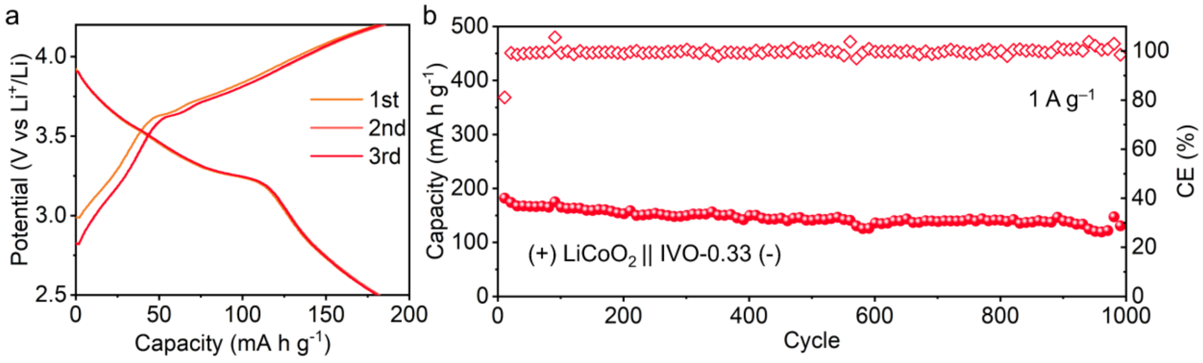

Figure S22. (a) Charge and discharge voltage profiles of (+) $LiCoO_2$ || IVO (-). (b) Cycling performance of (+) $LiCoO_2$ || IVO (-) at 1 A g^{-1}

Table S10. Comparison of lithium storage performances of IVO-0.33 and In-based materials.

		Current Density (A g^{-1})	Cycle	Capacity (mA h g^{-1})	Ref.
1	**This work**	1	613	1133.1	
2	**This work**	10	800	669.9	
3	**This work**	20	969	277.6	
4	**This work**	50	2500	222.1	
5	$In_{0.5}Zn_{0.5}O_{1.25}$	2	800	358	R1
6	In_2O_3@C	10	1000	282.2	R2
7	In_2O_3/HPNC	1	2000	623	R3
8	In_2O_3/3D graphene	0.05	100	770	R4
9	C/In_2O_3 nanosheets	0.4	400	782	R5
10	In_2O_3 NPs/Carbon fibre	0.1	500	435	R6
11	In_2O_3/C nanorods	1	1000	425	R7
12	InP@TiO_2-C	0.5	800	750	R8
13	In_2S_3/C	0.4	50	210	R9
14	In_2S_3 nanocrystals	0.5	100	329	R10

Ref.R1: *Appl. Surf. Sci.* **2019**, 470, 340.

Ref.R2: *Nano Energy* **2015**, 12, 339.

Ref.R3: *Appl. Surf. Sci.* **2020**, 513, 145894.

Ref.R4: *J. Mater. Chem. A* **2015**, 3, 18238.

Ref.R5: *Mater. Chem. Phys.* **2017**, 193, 89.

Ref.R6: *J. Alloy. Compd.* **2018**, 735, 319.

Ref.R7: *Mater. Chem. Phys.* **2017**, 193, 89.

Ref.R8: *Chem. Eng. J.* **2020**, 399, 125826.

Ref.R9: *J. Mater. Chem.* **2011**, 21, 18398.

Ref.R10: *CrystEngComm* **2016**, 18, 250.

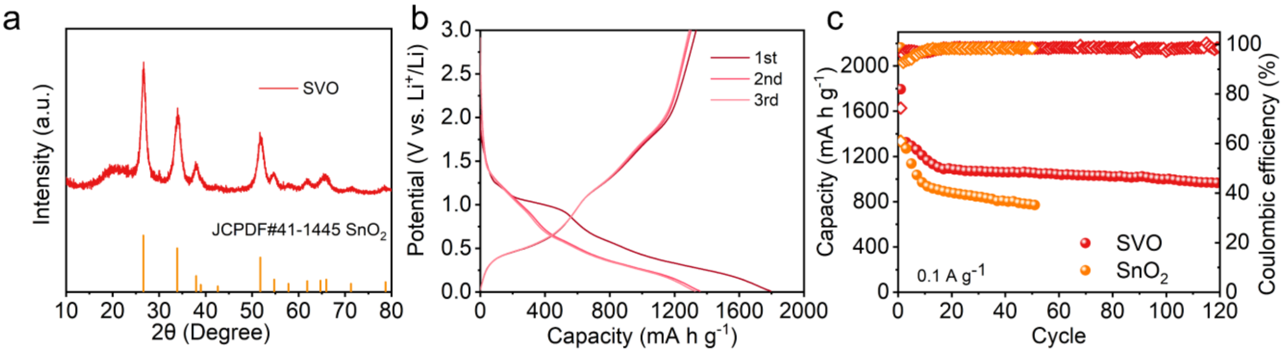

Figure S23. a) Powder XRD pattern of SVO. **b)** Charge and discharge curves of SVO at 0.1 A g^{-1}. **c)** Cyclic performance of SVO and SnO_2 at 0.1 A g^{-1}.

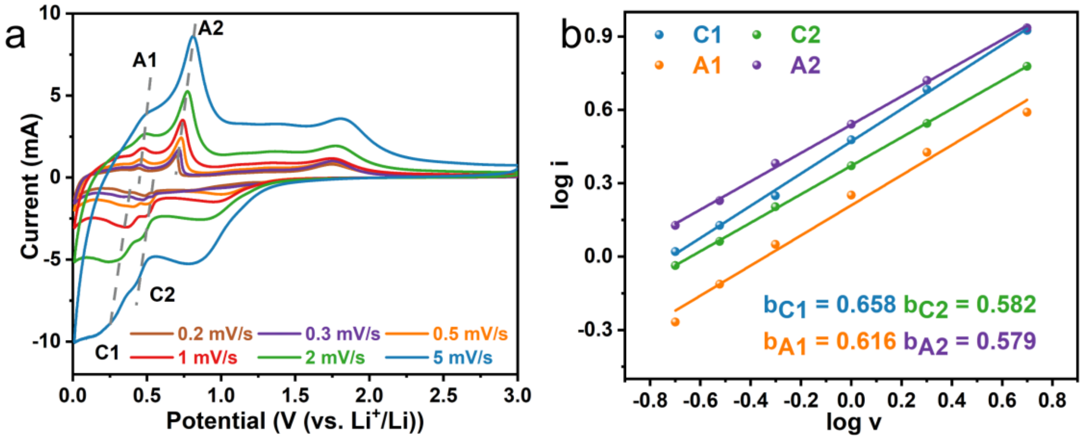

Figure S24. a) Cyclic voltammograms at varying sweep rates for In_2O_3 anodes versus. Li^+/Li. **b)** Plot of log i vs. log v of In_2O_3 shows the b-value is approximately 0.5, indicating a diffusion-controlled process.

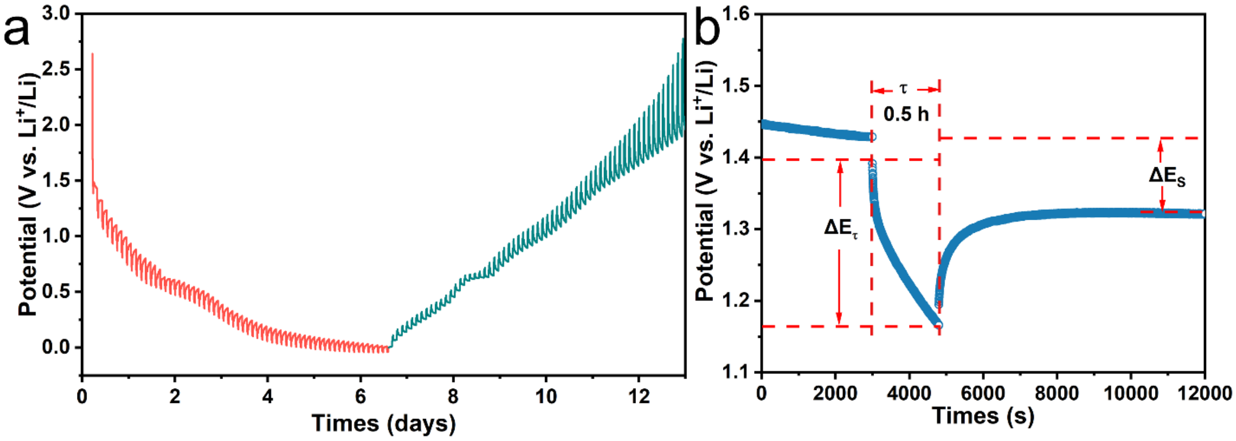

Figure S25. **a)** A single titration graph of IVO-0.33 during 2nd discharge cycle. **b)** Potential versus times plot displayed a GITT pattern.

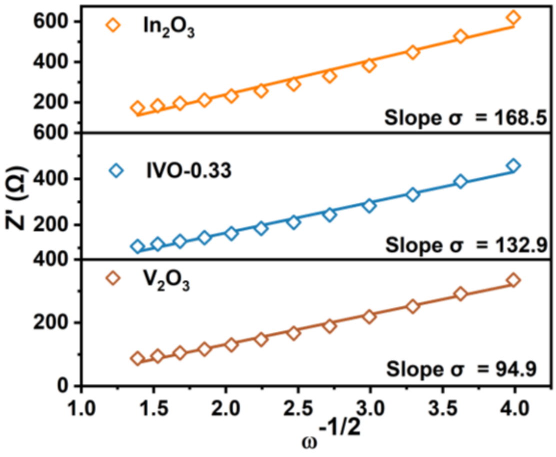

Figure S26. Real part of the impedance versus the square root of lower angular frequency ω of IVO-0.33, In_2O_3

and V_2O_3.

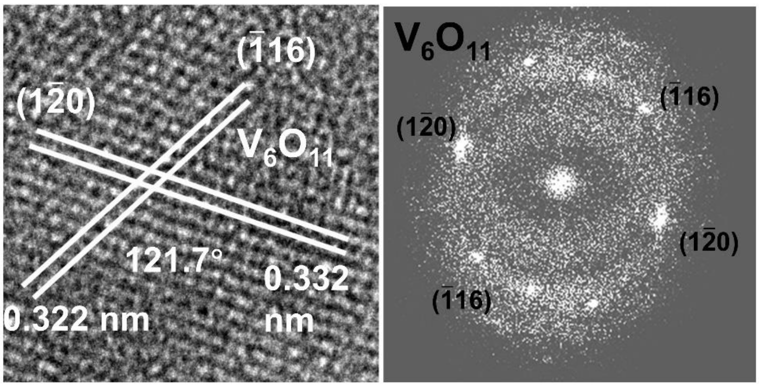

Figure S27. Lattice fringe of V_6O_{11} and corresponding FFT image of IVO-0.33 for discharge to 0.01 V after 10 cycles.

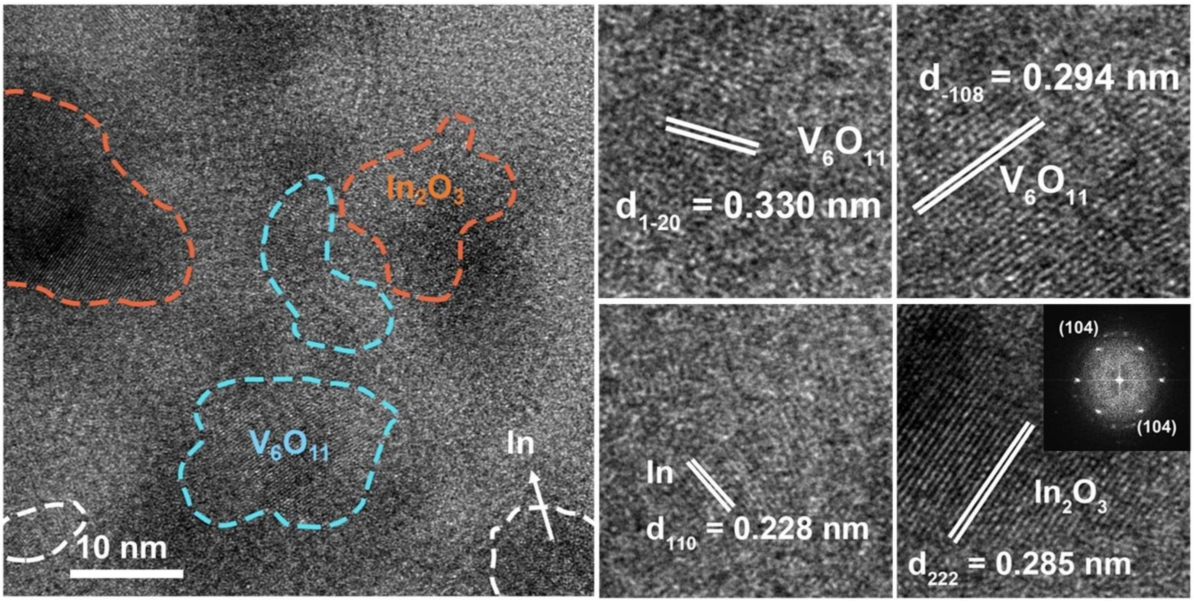

Figure S28. Ex-situ HRTEM, SAED and HADDF-STEM characterization of IVO-0.33 for charge to 3 V after 10 cycles, showing the lattice distances of V_6O_{11}, In and In_2O_3 (inset image displaying FFT of In_2O_3, displaying (104) lattice).

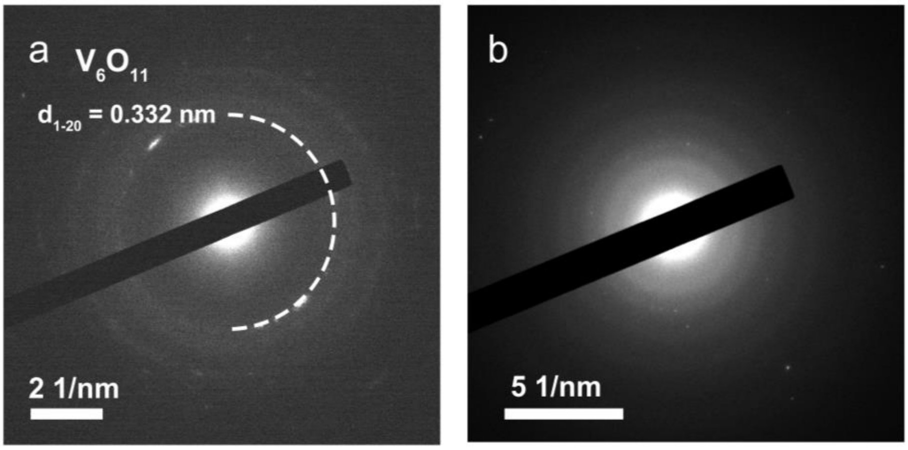

Figure S29. SEAD image of IVO-0.33 at **a)** 0.01 V and **b)** 3 V after 10 cycles.

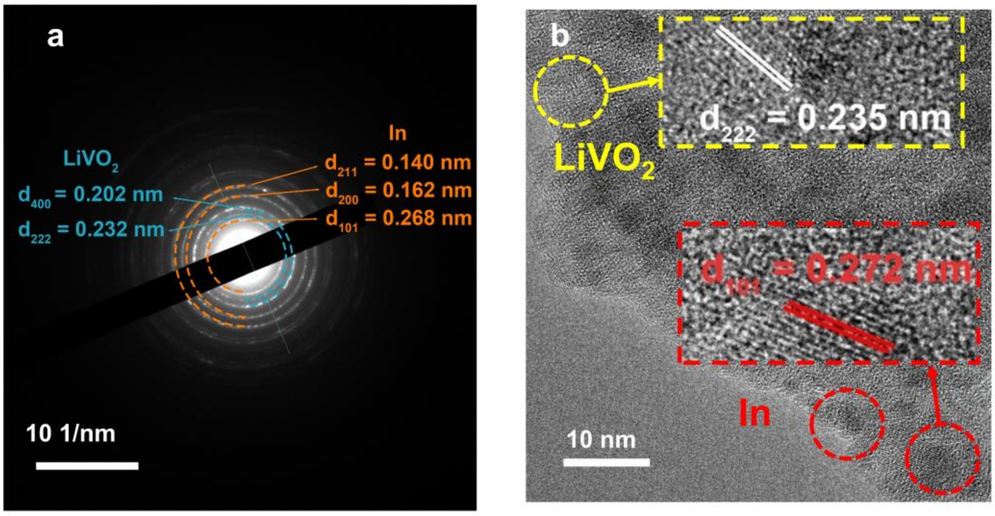

Figure S30. **a)** SEAD for IVO-0.33 after discharge to 1.5 V at 10th cycle, displaying the diffraction rings of In and cubic $LiVO_2$ (**JCPDF#36-0041**). **b)** Ex-situ HRTEM after discharge to 0.8 V at 10th cycle. The inset image displayed the lattice fringe of In and cubic $LiVO_2$.

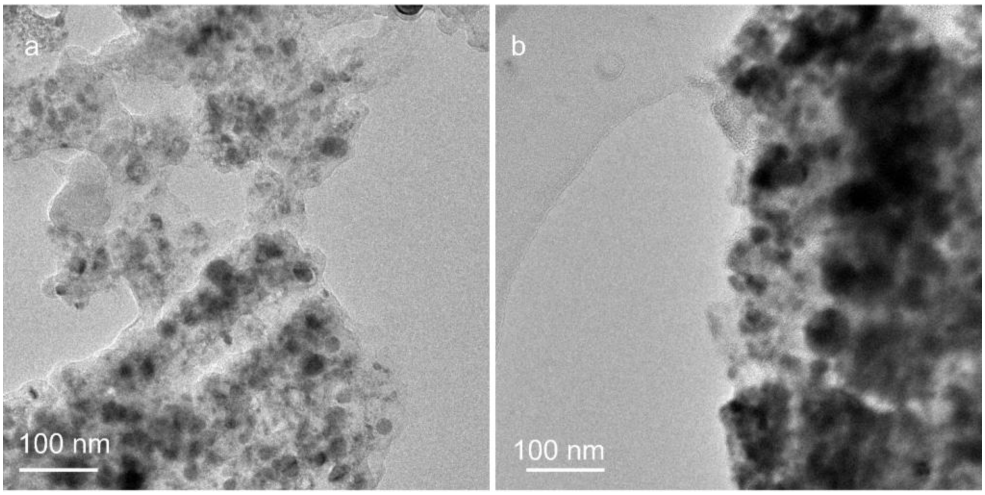

Figure S31. Low-resolution TEM images of **a)** IVO-0.33 and **b)** In_2O_3 charging to 3 V after 50 cycles.

References

[1] L. Zhou, K. Zhang, Z. Hu, Z. Tao, L. Mai, Y.-M. Kang, S.-L. Chou, J. Chen, *Adv. Energy Mater.* **2018**, 8, 1701415.

[2] L. Ji, Z. Lin, M. Alcoutlabi, X. Zhang, *Energy Environ. Sci.* **2011**, 4, 2682.

[3] H. Li, P. Balaya, J. Maier, *J. Electrochem. Soc.* **2004**, 151, A1878.

[4] Y. Luo, *CRC Press: Boca Raton, 2007.*

[5] K. Hemalatha, A. S. Prakash, G. K, M. Jayakumar, *J. Mater. Chem. A* **2014**, 2, 1757.

[6] T. Yuan, X. Yu, R. Cai, Y. Zhou, Z. Shao, *J. Power Sources* **2010**, 195, 4997.

[7] J. Zhou, N. Lin, L. Wang, K. Zhang, Y. Zhu, Y. Qian, *J. Mater. Chem. A* **2015**, 3, 7463.

[8] B. Ahmed, M. Shahid, D. H. Nagaraju, D. H. Anjum, M. N. Hedhili, H. N. Alshareef, *ACS Appl. Mater. Interfaces* **2015**, 7, 13154.

[9] B. Yan, X. Li, Z. Bai, M. Li, L. Dong, D. Xiong, D. Li, *J. Alloy. Compd.* **2015**, 634, 50.

[10] L. Wang, Y. Zhang, H. Guo, J. Li, E. A. Stach, X. Tong, E. S. Takeuchi, K. J. Takeuchi, P. Liu, A. C. Marschilok, S. S. Wong, *Chem. Mater.* **2018**, 30, 671.

[11] L. Yang, L. Liu, Y. Zhu, X. Wang, Y. Wu, *J. Mater. Chem.* **2012**, 22, 13148.

[12] L. Wu, J. Zheng, L. Wang, X. Xiong, Y. Shao, G. Wang, J. H. Wang, S. Zhong, M. Wu, *Angew. Chem. Int. Edit.* **2019**, 58, 811.

[13] J. Wang, Z. Liu, W. Yang, L. Han, M. Wei, *Chem Commun (Camb)* **2018**, 54, 7346.

[14] C. Hou, J. Wang, W. Du, J. Wang, Y. Du, C. Liu, J. Zhang, H. Hou, F. Dang, L. Zhao, Z. Guo, *J. Mater. Chem. A* **2019**, 7, 13460.

[15] K. Zhu, X. Wang, J. Liu, S. Li, H. Wang, L. Yang, S. Liu, T. Xie, *ACS Sustainable Chem. Eng.* **2017**, 5, 8025.

[16] T. Li, G. Nam, K. Liu, J.-H. Wang, B. Zhao, Y. Ding, L. Soule, M. Avdeev, Z. Luo, W. Zhang, T. Yuan, P. Jing, M. G. Kim, Y. Song, M. Liu, *Energy Environ. Sci.* **2022**, 15, 254.

[17] N. A. Chernova, M. Roppolo, A. C. Dillon, M. S. Whittingham, *J. Mater. Chem.* **2009**, 19, 2526.

[18] H. Ren, R. Yu, J. Qi, L. Zhang, Q. Jin, D. Wang, *Adv. Mater.* **2019**, 31, e1805754.

[19] S. Lou, X. Cheng, L. Wang, J. Gao, Q. Li, Y. Ma, Y. Gao, P. Zuo, C. Du, G. Yin, *J. Power Sources* **2017**, 361, 80.

[20] D. Chao, C. Zhu, X. Xia, J. Liu, X. Zhang, J. Wang, P. Liang, J. Lin, H. Zhang, Z. X. Shen, H. J. Fan, *Nano Lett.* **2015**, 15, 565.

[21] J. Ding, S. A. Abbas, C. Hanmandlu, L. Lin, C.-S. Lai, P.-C. Wang, L.-J. Li, C.-W. Chu, C.-C. Chang, *J. Power Sources* **2017**, 348, 270.

[22] Z. Yao, X. Xia, D. Xie, Y. Wang, C.-a. Zhou, S. Liu, S. Deng, X. Wang, J. Tu, *Adv. Funct. Mater.* **2018**, 28, 1802756.

[23] J. Come, V. Augustyn, J. W. Kim, P. Rozier, P.-L. Taberna, P. Gogotsi, J. W. Long, B. Dunn, P. Simon, *J. Electrochem. Soc.* **2014**, 161, A718.

[24] Z. Chen, C. Zhang, Z. Zhang, J. Li, *Phys. Chem. Chem. Phys.* **2014**, 16, 13255.

[25] H.-C. Chiu, X. Lu, J. Zhou, L. Gu, J. Reid, R. Gauvin, K. Zaghib, G. P. Demopoulos, *Adv. Energy Mater.* **2017**, 7, 1601825.

[26] Y. Sun, J. Wang, B. Zhao, R. Cai, R. Ran, Z. Shao, *J. Mater. Chem. A* **2013**, 1, 4736.
